# DEAD-box RNA helicase Dbp4/DDX10 is an enhancer of α-synuclein toxicity and oligomerization

**DOI:** 10.1371/journal.pgen.1009407

**Published:** 2021-03-03

**Authors:** Blagovesta Popova, Dan Wang, Christina Pätz, Dagmar Akkermann, Diana F. Lázaro, Dajana Galka, Miriam Kolog Gulko, Markus T. Bohnsack, Wiebke Möbius, Katherine E. Bohnsack, Tiago F. Outeiro, Gerhard H. Braus

**Affiliations:** 1 Department of Molecular Microbiology and Genetics, Institute for Microbiology and Genetics, University of Goettingen, Göttingen, Germany; 2 Department of Experimental Neurodegeneration, Center for Biostructural Imaging of Neurodegeneration, University Medical Center Goettingen, Göttingen, Germany; 3 Department of Molecular Biology, University Medical Center Goettingen, Göttingen, Germany; 4 Department of Neurogenetics, Electron Microscopy Core Unit, Max-Planck-Institute of Experimental Medicine, Göttingen, Germany; 5 Translational and Clinical Research Institute, Newcastle University, Newcastle upon Tyne, United Kingdom; University of Massachusetts Amherst, UNITED STATES

## Abstract

Parkinson’s disease is a neurodegenerative disorder associated with misfolding and aggregation of α-synuclein as a hallmark protein. Two yeast strain collections comprising conditional alleles of essential genes were screened for the ability of each allele to reduce or improve yeast growth upon α-synuclein expression. The resulting 98 novel modulators of α-synuclein toxicity clustered in several major categories including transcription, rRNA processing and ribosome biogenesis, RNA metabolism and protein degradation. Furthermore, expression of α-synuclein caused alterations in pre-rRNA transcript levels in yeast and in human cells. We identified the nucleolar DEAD-box helicase Dbp4 as a prominent modulator of α-synuclein toxicity. Downregulation of *DBP4* rescued cells from α-synuclein toxicity, whereas overexpression led to a synthetic lethal phenotype. We discovered that α-synuclein interacts with Dbp4 or its human ortholog DDX10, sequesters the protein outside the nucleolus in yeast and in human cells, and stabilizes a fraction of α-synuclein oligomeric species. These findings provide a novel link between nucleolar processes and α-synuclein mediated toxicity with DDX10 emerging as a promising drug target.

## Author summary

Neurodegenerative Parkinson’s disease affects about 2% of the over 65 years old human population. It is characterized by loss of dopaminergic neurons in midbrain and the presence of Lewy inclusion bodies that are predominantly composed of the α-synuclein protein. Expression of human α-synuclein in yeast cells results in dosage-dependent toxicity monitored as growth reduction and the formation of inclusions similar to mammalian neurons. Systematic analysis of yeast genes, which are essential for growth, revealed that reduced expression of central cellular proteostasis pathways, such as protein synthesis and ubiquitin-dependent protein degradation can enhance or reduce toxic effects of α-synuclein on yeast growth. Expression of α-synuclein affects not only early steps of ribosome biogenesis in yeast but also in human cells. We discovered the nucleolar DEAD-box RNA helicase Dbp4 as a novel strong enhancer of α-synuclein toxicity. The interaction of α-synuclein in yeast with Dbp4 as well as in human cells with its ortholog DDX10 results in sub-cellular exclusion from the nucleolus and promotes the accumulation of toxic oligomeric α-synuclein species. This molecular interaction of α-synuclein with DDX10 and its consequences for human cells provide a novel view in understanding the complexity of Parkinson’s disease.

## Introduction

Parkinson’s disease (PD) is a complex neurodegenerative disorder with diverse clinical features [1]. Neuronal loss in the *substantia nigra* and intracellular inclusions termed Lewy bodies (LB) are neuropathological hallmarks of PD. A major constituent of LB is the protein α-synuclein (αSyn) [2]. Misfolding and aggregation of αSyn plays a major role in PD pathogenesis [3]. Under pathological conditions, αSyn accumulates and can form oligomeric species that can further mature into different types of aggregated species [4,5]. Accumulating evidence suggests that oligomeric or protofibrillar forms of αSyn are responsible for neurotoxicity [6–8], which makes αSyn a key target for therapeutic development. The precise molecular events underlying αSyn neurotoxicity and factors that trigger its aggregation and pathogenicity remain elusive.

αSyn can localize in the nucleus of neuronal cells derived from PD patients and in cell and animal models expressing human αSyn. This may inhibit histone acetylation and promote neurotoxicity by inducing transcriptional deregulation [9–12]. The effects of αSyn in the nucleus are largely unknown. The PD-causing mutations A30P, A53T, and G51D in αSyn were shown to increase its nuclear accumulation [11,12]. Overexpression of A53T-αSyn in a mouse model results in its localization in nucleoli of dopaminergic neurons with different impacts on nucleolar activity [13]. Nucleolar dysfunction contributes to the pathology of several neurodegenerative disorders [14]. The nucleolus is the major site of rRNA production/processing and ribosome assembly, and nucleolar activity is tightly linked to the cellular well-being. Perturbation of nucleolar activity, defined as nucleolar stress, may occur at early disease stages. Dopaminergic neurons of PD brains reveal disrupted nucleolar integrity and increased nucleolar stress [15,16]. The association of impaired nucleolar activity with PD pathology is also supported by the interaction of nucleolin, an RNA-binding protein involved in ribosome biogenesis, with αSyn and DJ-1, two major proteins involved in PD [17,18]. Nucleolin levels were significantly reduced in *substantia nigra pars compacta* of PD brains [10,17]. Several hundred nucleolar proteins shuttle between different cellular compartments, and important regulatory functions can be altered by nucleolar disruption and release of nucleolar proteins to the nucleoplasm [19]. However, little is known about possible links between the regulators of nucleolar activity and αSyn species, particularly oligomers that contribute to the nucleolar stress in PD.

Proteotoxicity of αSyn was found in multiple cellular systems ranging from yeast to human [20]. As in neurons, expression of αSyn in *Saccharomyces cerevisiae* leads to the formation of inclusions and to a significant dose-dependent growth reduction [21–23]. Genome-wide screens with non-essential yeast mutant gene libraries enabled the discovery of multiple genes and cellular processes affecting αSyn-induced toxicity that were further validated in more complex model organisms [24–28]. In contrast, the regulatory roles of essential genes have rarely been explored. Essential genes exhibit a greater degree of conservation than non-essential genes between yeast and mammalian cells [29]. A systematic functional replacement of yeast essential genes by their human orthologs demonstrated that 47% of yeast genes could be successfully ‘humanized’, revealing the identical roles in both organisms [30].

We investigated essential pathways and genes that affect αSyn toxicity. Novel candidates were identified as modulators of αSyn toxicity by screening yeast growth profiles of two libraries comprising conditional alleles of essential genes. Most modulator genes encode proteins localized in the nucleus/nucleolus. *DBP4* was identified as strong enhancer of αSyn-induced toxicity and encodes the nucleolar DEAD-box RNA helicase Dbp4, which is required for ribosome biogenesis. Down-regulation of *DBP4* improved growth of yeast cells expressing αSyn, whereas overexpression of *DBP4* or its human ortholog *DDX10* dramatically exacerbated the growth inhibition. In yeast and human cells, αSyn caused mislocalization of Dbp4/DDX10 from the nucleolus and sequestered DDX10 into cytoplasmic inclusions. The results highlight *DDX10* as a promising novel drug target for intervention.

## Results

### Essential yeast genes modulate αSyn toxicity

Genome-wide screens using *S*. *cerevisiae* strain collections comprising conditional alleles of essential genes were performed to identify essential genes that modulate the cellular toxicity resulting from αSyn accumulation. The screening enabled the identification of negative modulators of αSyn toxicity, *i*.*e*. putative gene targets (evaluated by growth enhancement phenotype upon downregulation of the essential gene), as well as positive modulators *i*.*e*. protective genes (synthetic-sick phenotype upon downregulation of the essential gene). Yeast strains expressing αSyn-GFP were generated in the genetic background of two yeast collections. They comprise the yeast *Tet*-Promoters Hughes collection (yTHC) and the decreased abundance by mRNA perturbation (DAmP) collection. The yTHC collection contains 844 strains where the expression of the essential genes can be regulated by doxycycline [31]. The endogenous promoter of each essential gene is replaced with a *Tet*-titratable promoter in the genome. This promoter allows switching off the gene expression by addition of doxycycline to the yeast growth medium, resulting in protein depletion and allowing functional analyses. The DAmP collection comprises 842 strains expressing hypomorphic alleles of essential genes that exhibit modest growth defects [32]. The sequence encoding the 3’-untranslated region (3’ UTR) of each relevant gene is replaced by a kanamycin resistance cassette. This interruption destabilizes the corresponding transcript resulting in mRNA levels reduced 4- to 10-fold as compared to wild type.

**Fig 1:**
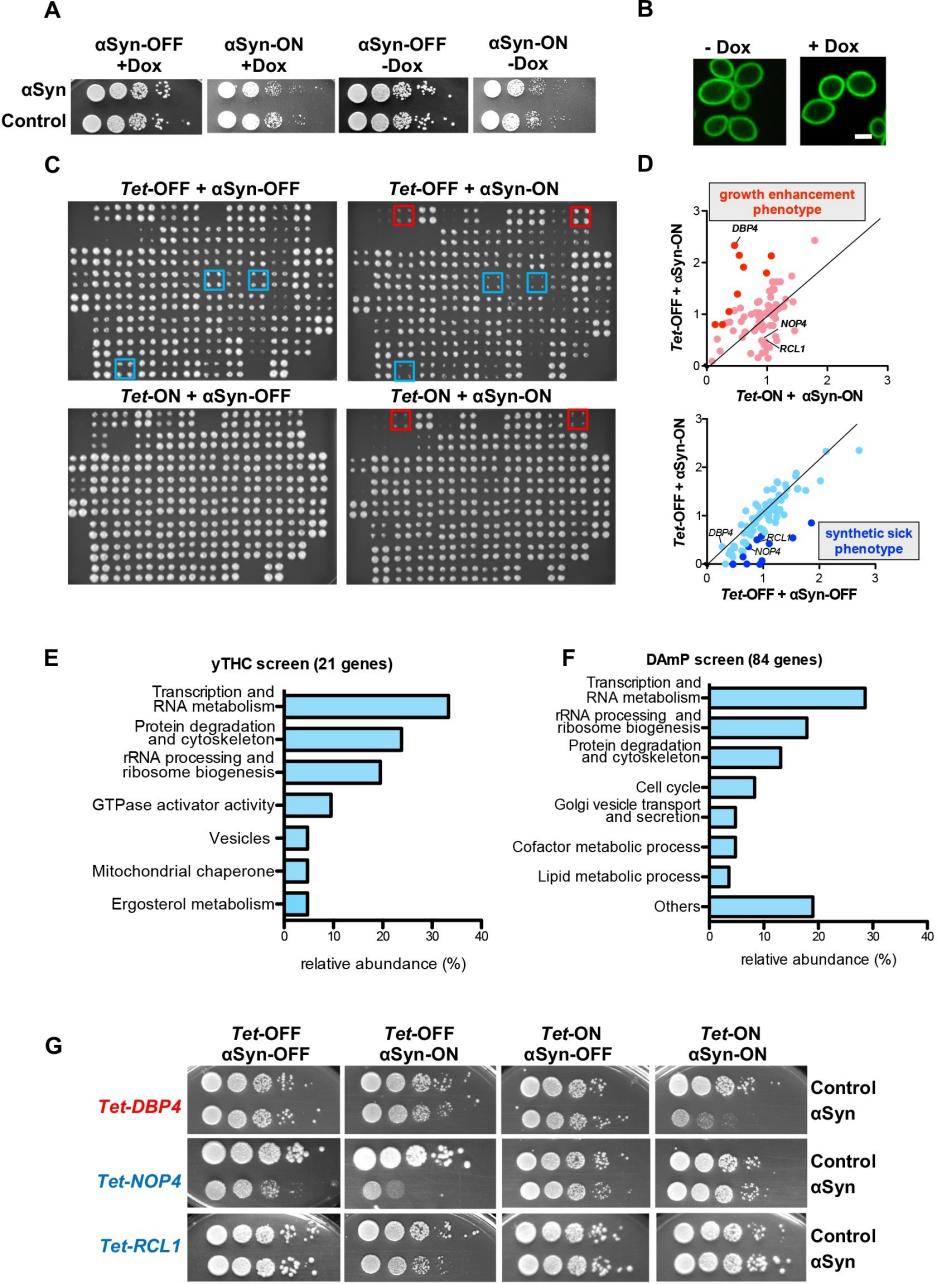
Yeast essential gene screens for modulators of αSyn toxicity. **(A)** Growth assays of yeast cells expressing *GAL1*-driven αSyn-GFP from two genomically integrated copies in Y7092 background, used as query strain in the *Tet*-Promoters Hughes collection (yTHC) and decreased abundance by mRNA perturbation (DAmP) collection screens, with empty vector as control. Cells were spotted in 10-fold dilutions on selective plates containing glucose (αSyn-OFF) or galactose (αSyn-ON), in presence (+) or absence (-) of 10 μg/ml doxycycline (Dox) that represses the *Tet*-promoter. **(B)** Fluorescence microscopy of the query strain after 6 h induction of αSyn-GFP expression in presence or absence of Dox. Scale bar = 2 μm. **(C)** Scoring of putative genetic interactions with essential genes from yTHC library. Example of growth phenotypes assayed in quadruplicate on selection plates with glucose (*Tet*-ON + αSyn-OFF), glucose + doxycycline (*Tet*-OFF + αSyn-OFF), galactose (*Tet*-ON + αSyn-ON) and galactose + doxycycline (*Tet*-OFF + αSyn-ON). Blue squares mark examples of colonies with inhibited growth upon *Tet-*promoter repression in presence of αSyn (synthetic sick phenotype–protective genes); red squares mark examples of colonies that grow better upon *Tet*-promoter repression in presence of αSyn (growth enhancement phenotype–target genes). **(D)** Relative growth rates estimated by the area of the individual colonies on the indicated plates relative to the control plate (*Tet*-ON + αSyn-OFF). Dark red: target genes; dark blue: protective genes. **(E-F)** Relative abundance of essential gene hits from yTHC **(E)** and DAmP screens **(F)** in functional categories. Percentage of essential genes associated with indicated Gene Ontology term is presented. **(G)** Growth effects on yeast cells upon interactions between αSyn and *Tet*-alleles of essential genes involved in ribosome biogenesis. Growth assays with *DBP4*, *NOP4* and *RCL1* mutant alleles, showing their ability to modify αSyn-induced toxicity compared to empty vector control.

Both libraries were crossed with the constructed yeast query strain RH3795, which harbored two copies of the *SNCA-GFP* gene stably integrated in Y7092 background under the regulatable *GAL1* promoter. Expression of αSyn from two gene copies in this strain is under the toxicity threshold. A spotting test of the query strain under inducing (galactose; αSyn-ON) and non-inducing (glucose; αSyn-OFF) conditions revealed that addition of doxycycline *per se* did not affect yeast growth (Fig 1A). Fluorescence microscopy demonstrated that αSyn did not form inclusions both in presence and absence of doxycycline (Fig 1A and 1B). Synthetic genetic array (SGA) technology was used for analysis of the genetic interactions [33]. The query strain was crossed to the arrayed mutant strains and then a robotic procedure was used for generation of double mutants. The double mutants were scored for growth defects relative to either of the single mutants. Genes that suppressed or enhanced the toxicity of αSyn were identified by comparison of the growth of yeast colonies upon down-regulation of essential gene expression (Fig 1C and 1D).

*Tet*-titratable promoter-dependent yTHC screen resulted in strong and robust genetic interactions that were analyzed and confirmed individually by random spore analyses (RSA) (S1 Fig) and spotting assays (S2 Fig). The stringent analyses confirmed 21 genetic interactions between αSyn and the yeast *Tet*-alleles of essential genes (S1 Table). Downregulation of the essential genes was accompanied by significant changes in αSyn inclusion formation for 71% of the analyzed genes (S3 Fig). Changes in aggregation and toxicity can be caused by differences in αSyn expression levels upon *Tet*-OFF which was individually tested for promising hits. As every reported interaction is confirmed by RSA and spotting assay, the false discovery rate is zero. Analysis of the mRNA perturbation DAmP screen and growth rate measurements of the colony size of double versus single mutants identified 84 mutant alleles that had a modest effect on αSyn-mediated growth retardation (S2 Table). All identified genes in both screens have described functions and 88% have annotated or predicted human homologs.

Genes were classified by functional category using Gene Ontology (GO) annotations, whereby each gene was classified according to one GO process (Fig 1E and 1F; S1 and S3 Tables). In both screens, the hits were clustered in the functionally related categories of transcription/RNA metabolism and ribosomal RNA (rRNA) processing/ribosome biogenesis (52% in yTHC and 47% in DAmP screen, respectively), as well as ubiquitin-dependent protein degradation/cytoskeleton organization (24% in yTHC and 13% in DAmP screen, respectively). Thus, most identified genes were involved in the central proteostasis pathways of protein synthesis and ubiquitin-dependent protein degradation. The majority (62%) of the encoded proteins revealed nuclear localization. Among them, the proteins encoded by the identified hits exhibited significant enrichment for nucleolar localization (p = 3.32 x 10^−5^ in yTHC and p = 3.58 x 10^−5^ in DAmP screen) (S4 and S5 Tables). This suggests a nucleolar contribution to the toxicity effect of αSyn on cellular growth.

A subset of three genes for nucleolar proteins involved in ribosome biogenesis identified in the yTHC screen were further analysed. They represent one putative drug target and two protective candidates (Fig 1G). Downregulation of the nucleolar *DBP4* as a drug target candidate improved growth of yeast cells expressing αSyn. Dbp4 is a DEAD box RNA helicase, required for early maturation events during biogenesis of the small ribosomal subunit [34,35]. The opposite effect was found for the protective genes *NOP4* and *RCL1* whose downregulation resulted in synthetic sick phenotypes. Nucleolar Nop4 protein is required for large ribosomal subunit assembly [36]. Rcl1 is an RNA 3’ phosphate cyclase, required for 18S rRNA biogenesis [37]. All three proteins are involved in ribosome synthesis but display opposite genetic interactions with αSyn.

### Dbp4 enhances αSyn-associated growth impairment

**Fig 2:**
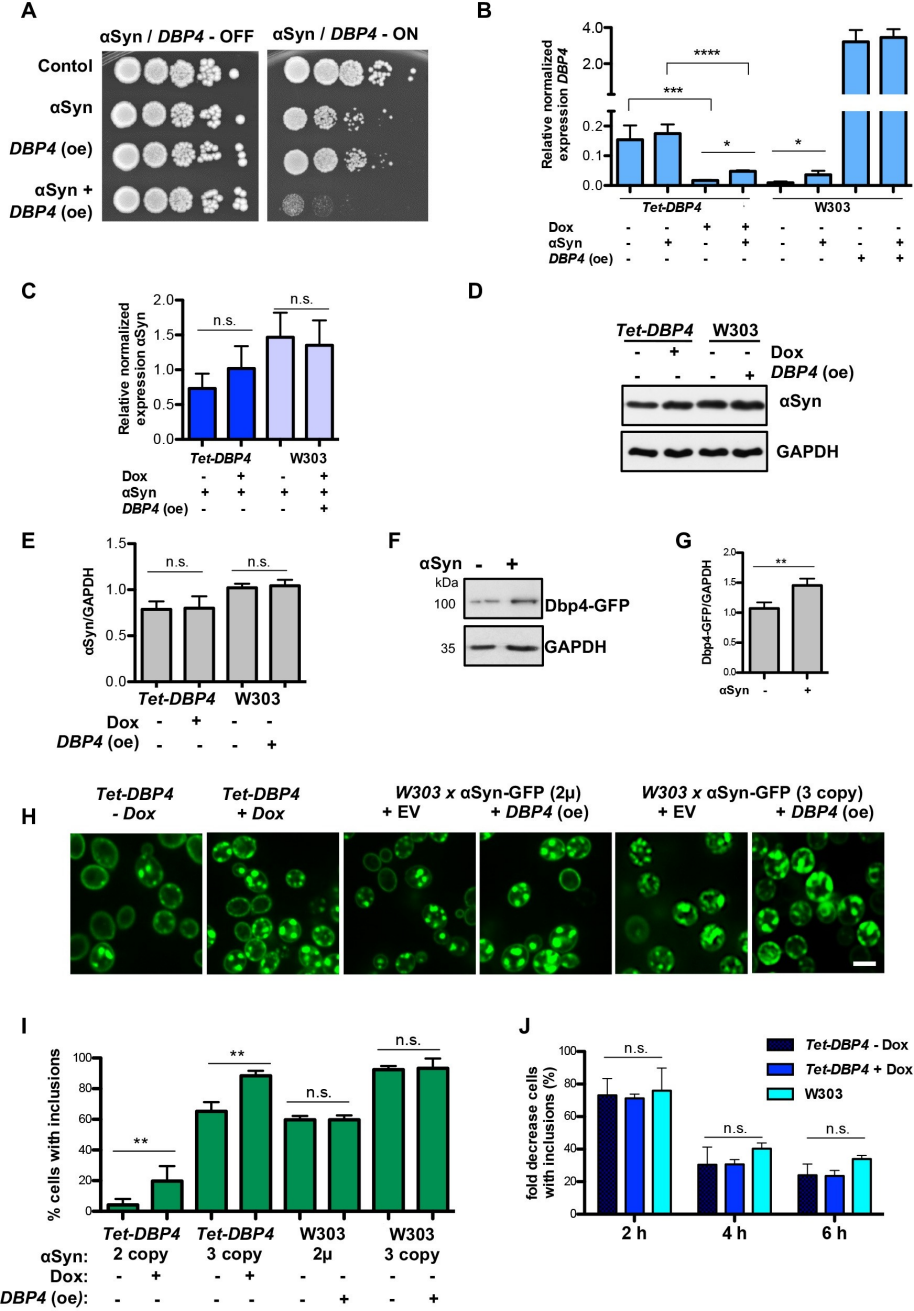
Dbp4 enhances αSyn-mediated growth impairment in yeast. **(A)** Growth assay of yeast cells expressing *GAL1*-driven αSyn-GFP from two genomic copies, either alone (αSyn) or co-expressed with *DBP4* (αSyn+*DBP4* oe). The isogenic background strain W303 was transformed with empty vectors (Control) or with 2μ plasmid overexpressing *DBP4* (*DBP4* oe). **(B)** Relative mRNA expression level of *DBP4* in *Tet*-*DBP4* and W303 strains determined by qRT-PCR in presence or absence of αSyn expression. Addition of doxycycline (Dox) efficiently downregulates the expression of *Tet*-*DBP4*. Expression of αSyn upregulates the mRNA levels of *Tet-DBP4* upon downregulation of the *Tet*-promoter or of natively expressed *DBP4* in wild type W303. Overexpression of *DBP4* increases considerably the mRNA levels. Significance of differences was calculated with t-test (*p < 0.05; ***p < 0.001; ****p < 0.0001, n = 4). **(C)** Relative abundance of αSyn mRNA from three gene copies in *Tet*-*DBP4* and from 2μ vector in W303 strains, determined by qRT-PCR. Significance of differences was calculated with t-test (n.s.; n = 4). **(D)** Western blot analysis of αSyn protein levels of cells from (C) with GAPDH antibody used as loading control. **(E)** Densitometric analysis of the immunodetection of αSyn relative to GAPDH loading control. Significance of differences was calculated with t-test (n.s.; n = 3). **(F)** Western blot analysis of Dbp4-GFP protein levels of cells expressing *DBP4*-GFP from native promoter with GAPDH antibody used as a loading control. **(G)** Densitometric analysis of the immunodetection of Dbp4-GFP relative to GAPDH loading control. Significance of differences was calculated with t-test (**p < 0.01; n = 3). **(H)** Fluorescence microscopy of yeast cells expressing αSyn-GFP from three gene copies in *Tet*-*DBP4* strain in the presence (+) or absence (-) of doxycycline (left panels); from a 2μ vector in W303 strain (middle panels) or from three gene copies of αSyn-GFP in W303 strain (right panels) 6 h after induction of expression in galactose-containing medium. *DBP4* was overexpressed from 2μ plasmid. EV = empty vector. Scale bar = 5 μm. **(I)** Quantification of the percentage of cells displaying αSyn-GFP inclusions in strains from H. Significance of differences was calculated with t-test (**p < 0.01, n = 4). **(J)** Promoter shut-off of cells from (H). Cells with inclusions were counted up to 6 h after *GAL1*-promoter shut-off and normalized to time point zero. Significance of differences was calculated with One-way Anova test (n.s.; n = 3).

The effect of overexpression of *DBP4* on αSyn toxicity was assessed in yeast wild type W303 background. High copy number expression of *DBP4* resulted in dramatic enhancement of the growth inhibition of the αSyn strain with two αSyn-encoding gene copies that normally exhibits slight toxicity (Fig 2A). In contrast to *DBP4*, overexpression of *RCL1* rescued the growth retardation of yeast strain with three αSyn-encoding gene copies. Expression of αSyn from three gene copies leads to increased protein level and causes high toxicity [22]. This confirms *RCL1* as protective gene against αSyn damage. *NOP4* overexpression had no impact on αSyn toxicity (S4A and S4B Fig).

The levels of *DBP4* expressed from its native promoter or under conditions of downregulation of the *Tet-*promoter were compared (Fig 2B). qRT-PCR confirmed that addition of doxycycline efficiently downregulates the mRNA level of *DBP4* in the *Tet-DBP4* strain. Notably, αSyn upregulated the expression of *DBP4* in *Tet-DBP4* strain in the presence of doxycycline and in W303 wild type strain upon normal expression level. αSyn did not affect *DBP4* mRNA levels in cells overexpressing *DBP4*. *DBP4* expression from *Tet-DBP4* allele was significantly higher than the endogenous *DBP4* expression in wild type W303 strain. Therefore, we assessed whether downregulation of *DBP4* levels below normal would similarly reduce αSyn toxicity. We made use of DAmP strain with reduced *DBP4* mRNA levels (S4C Fig). Growth assays revealed slightly improved growth of yeast cells expressing αSyn in DAmP strain in comparison with the growth phenotype in the isogenic BY4741 wild type yeast strain (S4D Fig). These results corroborate our previously observed findings (Fig 2A) that the αSyn toxicity is directly correlated with increased levels of *DBP4* expression and can be partially rescued if the essential gene is downregulated.

We next analyzed the effect of overexpression of *DBP4* in cells expressing *GAL1-*driven A30P variant of αSyn that has different toxicity properties to those of wild type αSyn. A30P-αSyn forms only inclusions when highly expressed without causing yeast growth inhibition because the aggregation of A30P is only transient [22,38]. Overexpression of *DBP4* impaired growth of A30P-αSyn expressed from three copies or overexpressed from 2μ vector (S5A Fig). This suggests an effect of Dbp4 on A30P-αSyn toxicity and corroborates its role as αSyn toxicity enhancer.

As αSyn toxicity is dependent on its expression levels [21,22], we examined whether αSyn toxicity upon differential expression of *DBP4* is connected with changes in αSyn expression. The expression of αSyn was not significantly changed upon downregulation or overexpression of *DBP4* (Fig 2C). Immunoblotting analysis showed no significant differences in the steady-state protein levels of αSyn (Fig 2D and 2E) or A30P-αSyn (S5C and S5D Fig) after 6 h of expression excluding the possibility that differences in toxicity are due to differences in αSyn levels. The protein levels of Dbp4-GFP were examined under its native promoter in absence and presence of αSyn expression. The protein levels corresponded to the changes in mRNA levels and were increased upon αSyn expression (Fig 2F and 2G).

Fluorescence microscopy was used to assess whether the *DBP4*-dependant αSyn growth inhibition is accompanied by changes in αSyn inclusion formation. Surprisingly, downregulation of *Tet-DBP4* expression in yeast strains with two or three genomically integrated copies of αSyn-GFP resulted in increased numbers of cells with inclusions (Fig 2H and 2I). However, overexpression of *DBP4* in wild type strain did not affect the inclusion formation. Similarly, high copy expression of *DBP4* did not affect the aggregate formation of A30P (S5D and S5E Fig). The clearance of αSyn inclusions was also assessed because inefficient clearance may lead to accumulation of toxic protein species resulting in cytotoxicity. Promoter shut-off studies were performed where αSyn expression was induced for 4 h in galactose-containing medium, followed by promoter shut-off in glucose-containing medium that represses the *GAL1* promoter from which αSyn is expressed (Fig 2J). The clearance of αSyn inclusions was not affected by the expression levels of Dbp4 up to 6 h after promoter shut-off.

This suggests that Dbp4-dependent enhancement of αSyn cytotoxicity is not accompanied with increased αSyn inclusion formation, and that toxicity and aggregation are distinct outcomes. αSyn toxicity directly correlates with *DBP4* expression levels and can be rescued by downregulation of the essential gene.

### Levels of pre-rRNA intermediates are altered in αSyn-expressing cells

**Fig 3:**
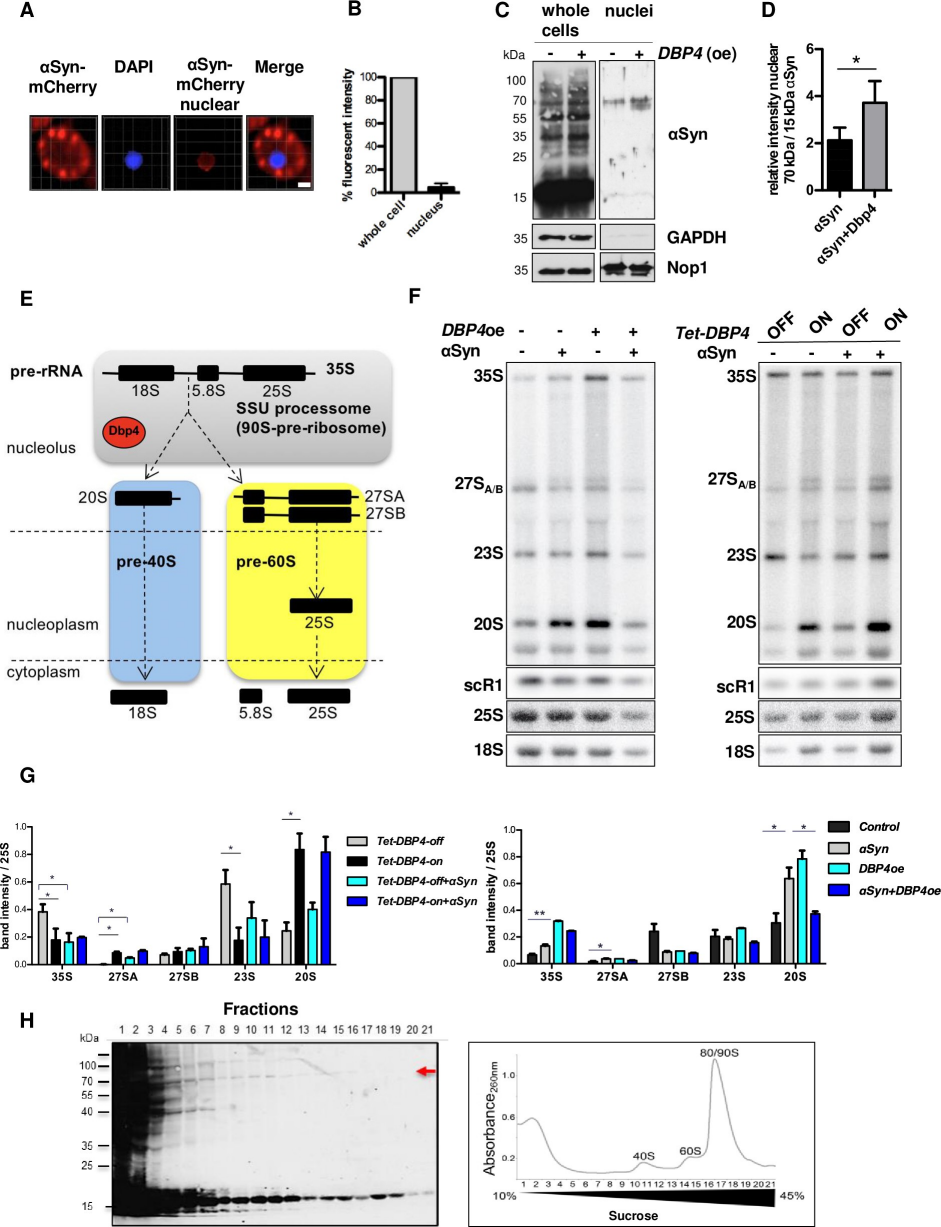
αSyn affects pre-rRNA processing in yeast. **(A)** Live-cell microscopy of yeast cells expressing *GAL1*-driven αSyn-mCherry from a 2μ vector after 6 h of protein expression. Nuclei were stained with DAPI and nuclear segmentation was performed with SlideBook 6.0 software. Scale bar = 1 μm. **(B)** Relative amount of the nuclear subpopulation of αSyn-mCherry evaluated from red fluorescence intensity of the nucleus as % of total red fluorescence intensity per cell (n = 30). **(C)** Immunoblotting of nuclear fractions and whole cell extracts of yeast cells expressing αSyn using an αSyn antibody. GAPDH antibody was used as control for nuclear fraction purity, and Nop1 antibody as nuclear marker. **(D)** Densitometric analysis of relative abundance of αSyn oligomeric to monomeric species. Significance of differences was calculated with t-test (*p < 0.05; n = 4). **(E)** Schematic diagram of ribosome assembly and the major steps of pre-rRNA processing in yeast. The initial 35S pre-rRNA transcript is processed to mature the 18S, 5.8S and 25S rRNAs via various pre-rRNA intermediates (20S and 27S) in a stepwise process depicted by dashed arrows. The colored boxes depict pre-ribosomal particles: grey– 90S/SSU processome, orange–pre-40S particles, blue–pre-60S particles. **(F)** Total RNA was extracted from cells expressing (+) or not (-) αSyn. *DBP4* was overexpressed from 2μ vector in W303 strain (left panel) or in the *Tet*-*DBP4* strain in the presence (*Tet*-OFF) or absence (*Tet*-ON) of doxycycline (right panel). RNAs were separated by denaturing agarose gel electrophoresis, and transferred to a nylon membrane. Northern blotting with probes hybridizing in the internal transcribed spacers 1 and 2 detected 20S, 23S, 27SA, 27SB, and 35S pre-rRNAs. The mature 18S and 25S rRNA were detected with specific hybridization probes. scR1 (small cytoplasmic RNA 1) transcript from the signal recognition particle was used as a loading control. **(G)** Densitometric analysis of the detected pre-rRNA species relative to 25S rRNA. Significance of differences was calculated with t-test (*p < 0.05; **p < 0.01). Cells transformed with empty vector were used as a control. **(H)** αSyn co-sediments with pre-ribosomal complexes. Whole yeast extracts from cells expressing αSyn were separated by sucrose density gradient centrifugation. The absorbance of each fraction at 260 nm was used to generate a profile of the ribosomal complexes. 21 fractions were collected from the top of the gradient. Proteins precipitated from each gradient fraction were analyzed by immunoblotting with αSyn antibody. Red arrow: αSyn oligomeric species (~70 kDa).

αSyn subcellular localization was analyzed to examine whether the impact of nucleolar proteins on αSyn toxicity is related to the presence of αSyn subpopulation within the nucleus. Fluorescence microscopy was applied to cells expressing αSyn-mCherry, which were segmented using SlideBook 6.0 software. A small nuclear αSyn population could be verified (Fig 3A and 3B). The presence of αSyn species in the nucleus was further characterized biochemically. Nuclear extracts from yeast cells expressing non-tagged αSyn were prepared and resolved by SDS gel electrophoresis. Immunodetection revealed a nuclear fraction of αSyn oligomers with molecular weight of ~70 kDa in addition to the weaker band at ~15 kDa, corresponding to monomeric αSyn (Fig 3C). The purity of the nuclear fraction was confirmed by the presence of the Nop1 nucleolar marker and lack of the cytoplasmic protein Glycerinaldehyd-3-phosphat-Dehydrogenase (GAPDH). Nuclear αSyn localization was affected by Dbp4 because overexpression of *DBP4* increased the ratio of oligomeric αSyn compared to the monomeric form in the nucleus (Fig 3D).

Dbp4 is an RNA helicase involved in early stages of ribosome biogenesis as a component of the small subunit processome (SSU processome), which is a large ribonucleoprotein (RNP) complex containing numerous ribosomal proteins and *trans*-acting ribosome assembly factors (Fig 3E) [39]. Dbp4 is essential for 18S rRNA production. Depletion of Dbp4 or substitution of key residues within the catalytic site impairs pre-rRNA early cleavages and causes accumulation of the U14 small nucleolar RNA (snoRNA) on pre-ribosomal complexes [34]. Therefore, we examined whether the toxicity enhancement effect of *DBP4* overexpression is dependent on its helicase activity. Two Dbp4 variants were analyzed with a substitution in motif I that strongly impairs ATPase activity (GKT to GRT substitution) or amino acid exchanges in motif III (SAT) that affect the coupling of ATP hydrolysis to the helicase activity (S225A/T227A double substitution). Overexpression of the two mutant alleles resulted in similar severe growth retardation in the presence of αSyn as overexpression of wild type Dbp4 (S6 Fig). This suggests that Dbp4 enzymatic activity does not contribute to the toxicity enhancement phenotype upon overexpression. However, the Dbp4 enzymatic activity might have an impact on αSyn toxicity at physiological protein concentrations.

Next, it was assessed whether expression of αSyn interferes with processing of pre-rRNAs. Analysis of steady-state pre-rRNA levels in W303 cells expressing or not αSyn by northern blotting using probes hybridizing to different pre-rRNA regions revealed significant accumulations of the 35S, 27SA and 20S pre-rRNA transcripts in the presence of αSyn in comparison to the control (Fig 3F left panel and 3G left panel). Increased levels of Dbp4 upon overexpression from 2μ vector led to accumulation of the 35S and 20S pre-rRNA species. It has been observed previously that overexpression of ribosome biogenesis factors can lead to defects in pre-rRNA processing. As both the individual overexpression of Dbp4 or αSyn lead to 20S accumulation, it is interesting that the combined overexpression of these two factors showed normal levels of 20S pre-rRNA. Overexpression of either αSyn or Dbp4 had no significant effect on the ratio of the 25S to 18S rRNA or their level relative to an independent loading control scR1 (S6C Fig). Expression of *DBP4* from the *Tet*-promoter rather than its endogenous one causes increased expression level (Fig 2B). Depletion of Dbp4 (*Tet-DBP4*-off) caused accumulation of 35S pre-rRNA and 23S pre-rRNAs and decreased levels of 27SA and 20S pre-rRNA (Fig 3F right panel and 3G right panel), as well as the mature 18S rRNA (S6D Fig), in accordance with published results [34,35,40,41]. In this strain, in the presence of overexpressed Dbp4 (*Tet-DBP4*-ON), expression of αSyn did not significantly alter the levels of any pre-rRNA intermediates. Notably, in the presence of αSyn, depletion of Dbp4 had a milder effect on pre-rRNA processing than in cells not expressing αSyn (Fig 3F right panel and 3G right panel). These results suggest that αSyn expression affects the levels of pre-rRNA intermediates and that there is interplay between the expression of αSyn and Dbp4. We therefore tested whether αSyn species co-sediment with (pre-)ribosomal complexes in sucrose density gradients. The distribution of αSyn across the gradient revealed the existence of monomeric αSyn, sedimenting with high-molecular weight fractions, suggesting existence SDS-soluble high molecular weight αSyn species (Fig 3H). Three SDS-stable oligomeric αSyn species with molecular weights of ~40 kDa, ~70 kDa and ~100 kDa were also detected. The 70 kDa species partly co-migrated with pre-40S, pre-60S and 90S pre-ribosomal complexes. The 70 kDa species had the same mobility as the identified SDS-stable oligomers in the nucleus and partly co-migrated with pre-40S, pre-60S and 90S pre-ribosomal complexes. These oligomers or the monomeric αSyn that also sediments with the high-molecular weight fractions may associate with pre-ribosomal complexes and thus interfere with pre-rRNA processing.

**Fig 4:**
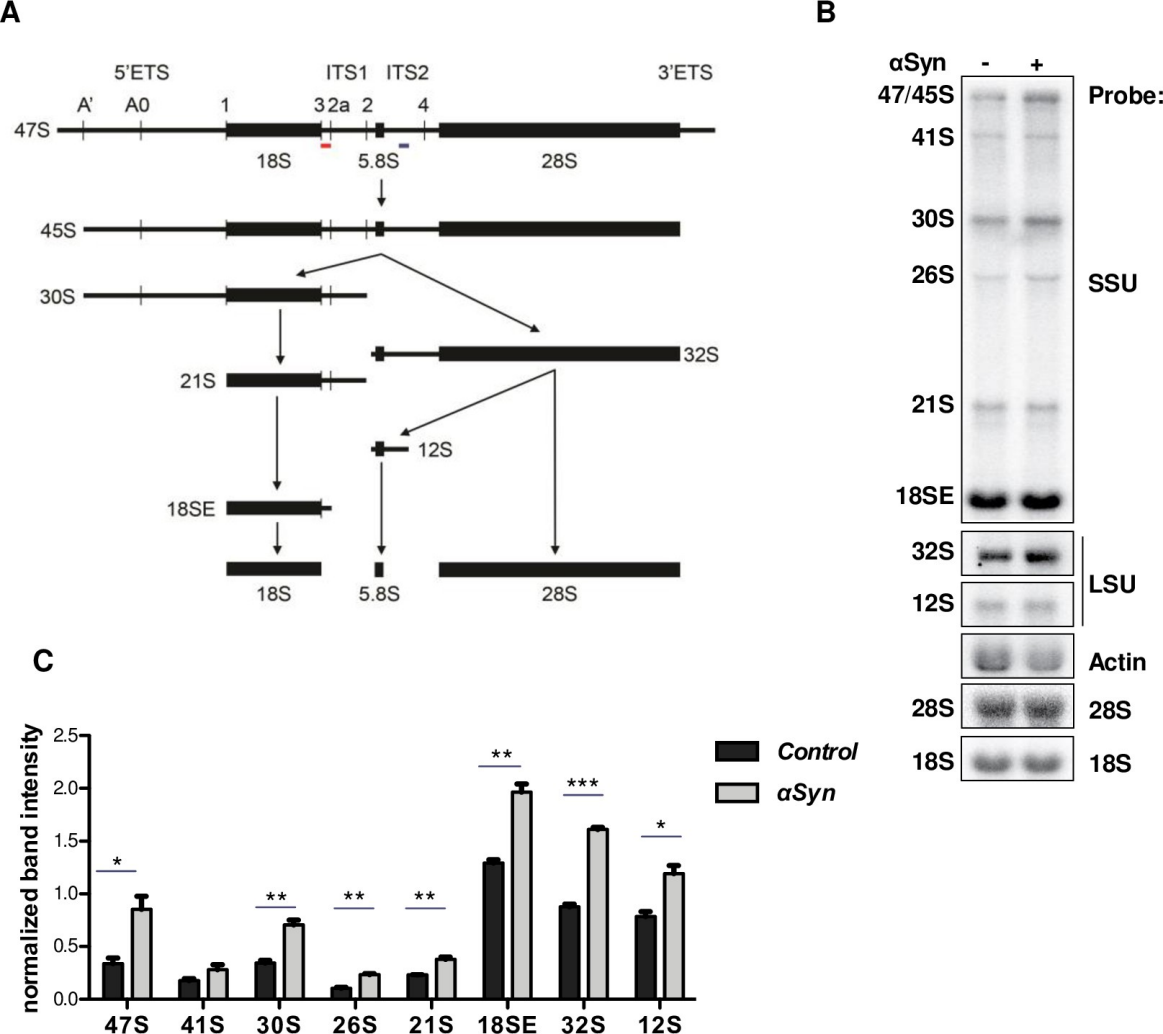
αSyn causes accumulation of pre-rRNA intermediates in human cells. **(A)** Schematic view of the major pre-rRNA intermediates in human cells. The initial 47S pre-rRNA transcript is processed to mature the 18S, 5.8S and 28S rRNAs via various pre-rRNA intermediates. The position of the SSU probe and LSU probe used for northern hybridization are indicated by red and blue lines respectively. ITS: internal transcribed spacer; ETS: external transcribed spacer. **(B)** Total RNA was extracted from HEK293 cells stably transfected with EGFP (control) or αSyn-EGFP. RNAs were separated by denaturing agarose gel electrophoresis and transferred to a nylon membrane. The mature 18S and 28S rRNA were visualized using specific hybridization probes. Northern blotting was performed with a probe hybridizing to the 5′ end of ITS1 (SSU probe) for detection of 18S rRNA precursors, or with a probe hybridizing to ITS2 (LSU probe) for detection of 28S and 5.8S rRNA precursors. Actin transcript was used as a loading control. **(C)** Densitometric analysis of band intensities of the indicated transcripts relative to the actin loading control. Significance of differences was calculated with t-test (*p < 0.05; **p < 0.01; ***p < 0.001; n = 3).

Next, we examined whether αSyn expression has an effect on pre-rRNA processing in human cells. Human Embryonic Kidney 293 (HEK) cells were used that express EGFP as a control or αSyn-EGFP. Analysis of pre-rRNA levels by northern blotting was performed using probes hybridizing to pre-rRNA regions of transcripts on the pathway of 18S rRNA maturation (SSU probe) or 28S rRNA maturation (LSU probe) (Fig 4A and 4B). The results revealed significant increase of the levels of 47S, 30S, 26S and 21S pre-rRNAs (18S precursors), as well as 32S and 12S pre-rRNAs (28S and 5.8S precursors) in presence of αSyn (Fig 4C), and no significant effect on the ratio of 28S to 18S rRNA (S6E Fig). These data support our findings from yeast cells that αSyn affects pre-rRNA levels.

### αSyn interacts with Dbp4 and causes its mislocalization to the nucleoplasm and cytoplasmic inclusions

**Fig 5:**
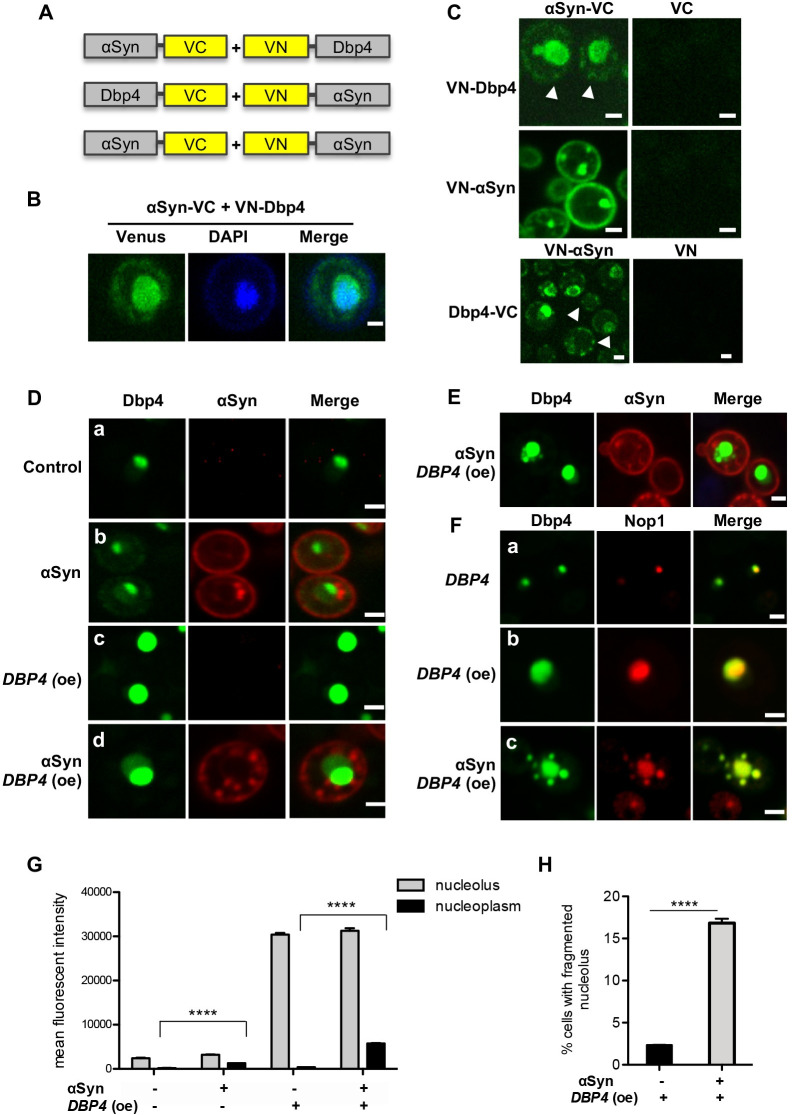
Dbp4 physically interacts with αSyn. **(A)** Schematic representation of Bimolecular Fluorescence Complementation assay (BiFC) constructs. Dbp4 and αSyn were fused to the non-fluorescent complementary N- and C-terminal fragments of fluorescent Venus reporter protein (VN and VC) in two different fusion protein combinations. (**B)** Live-cell fluorescence microscopy of yeast W303 cells expressing VN-Dbp4 and αSyn-VC constructs with DAPI stained nucleus. Scale bar = 1 μm **(C)** BiFC with different combinations of the fusion constructs. VN-αSyn + αSyn-VC served as positive and BiFC with VC or VN as negative controls. The image display is scaled to the minimum and maximum pixel intensity values per image for optimal noise-to signal ratios. Scale bar = 1 μm. White arrow heads indicate cytoplasmic foci. **(D)** Fluorescence microscopy of cells expressing *DBP4*-GFP from its native promoter with empty vector as a control (a) or αSyn-mCherry (b). Overexpression of *DBP4*-GFP from 2μ vector with empty vector as a control (c) or with αSyn-mCherry (d). αSyn expression leads to Dbp4-GFP mis-localization from nucleolus (bright green fluorescence) to nucleoplasm (faint green fluorescence). Scale bar = 1 μm. **(E)** Representative example of fragmented nucleolar phenotype observed upon overexpression of *DBP4*-GFP in presence of αSyn. Scale bar = 1 μm. **(F)** Colocalization of Dbp4 with the nucleolar marker Nop1. Cells expressing *DBP4*-GFP from its native promoter (a); overexpressing *DBP4*-GFP from 2μ vector (b); or overexpressing *DBP4*-GFP and αSyn (no tag) from 2μ vectors (c) were co-expressed with *NOP1*-RFP from single-copy vector. Fluorescence microscopy reveals colocalization of Dbp4-GFP and Dbp4-GFP foci with the nucleolar marker Nop1-RFP. Scale bar = 1 μm. **(G)** Quantification of the GFP mean fluorescence intensity in the nucleolus und nucleoplasm (nucleus excluding nucleolar region) of cells from (D). Significance of differences was determined with t-test (****p < 0.0001; n = 60). **(H)** Quantification of the percentage of cells displaying fragmented nucleoli upon overexpression of *DBP4*-GFP with t-test (****p < 0.0001; n = 200).

Bimolecular Fluorescence Complementation assay (BiFC) was performed to analyze whether the functional consequences of αSyn expression were due to a physical interaction with Dbp4. αSyn and Dbp4 were fused to the non-fluorescent complementary N- and C-terminal fragments of the fluorescent reporter protein Venus (VN and VC) (Fig 5A). Two different fusion protein combinations were constructed and tested for bimolecular fluorescence complementation. Co-expression of αSyn-VC + VN-Dbp4 constructs in yeast yielded green fluorescence indicating reconstitution of the Venus fluorophore by the interaction of Dbp4 with αSyn. The fluorescent signal was localized in the nucleus and in cytoplasmic foci near the plasma membrane that resemble the inclusions formed by αSyn alone (Fig 5B and 5C). Co-expression of VN-αSyn + Dbp4-VC revealed the same ability to reconstitute the Venus fluorophore with similar subcellular localization of the signal (Fig 5C). We tested the specificity of the interaction between Dbp4 and αSyn with competition assays. The BiFC constructs were expressed in presence or absence of non-tagged αSyn (S7A Fig), with αSyn BiFC as a control. Expression of non-tagged αSyn could compete the fluorescent signal (S7A and S7B Fig), suggesting that the interactions between Dbp4 and αSyn are specific. Western blot analysis confirmed the expression of all constructs in yeast cells (S7C Fig). We quantified the efficiency of the fluorescence complementation between VN-αSyn + Dbp4-VC and compared it to VN-αSyn + αSyn-VC (S7D Fig). The BiFC fluorescence signal intensity per cell was ~8.5-fold lower for VN-αSyn + Dbp4-VC, suggesting that only a small fraction of Dbp4 interacts with αSyn in yeast cells.

The subcellular localization of Dbp4-GFP was assessed. Dbp4-GFP expressed from its native promoter was localized exclusively in the nucleolus, as expected (Figs 5D and S8A). Upon expression of αSyn-mCherry, a faint fluorescence signal in the GFP channel was observed outside of the nucleolus (Fig 5D and 5G). This effect appeared specific for Dbp4 as expression of αSyn-mCherry did not change the subcellular localization of the nucleolar Rcl1-GFP or Nop4-GFP (S8B and S8C Fig). The mislocalization of Dbp4 to the nucleoplasm was increased in cells overexpressing Dbp4-GFP and αSyn-mCherry. While overexpression of Dbp4-GFP alone resulted in an increased but exclusively nucleolar signal, co-expression with αSyn-mCherry resulted in significant GFP staining in the nucleoplasm outside the nucleolus. We could not observe co-localization of αSyn-mCherry cytoplasmic foci and Dbp4-GFP with regular co-localization microscopy, which might be due to the small fraction of Dbp4, interacting with αSyn and leaving the nucleus.

A loss of nucleolar integrity was observed in 16.8% of the cells co-expressing Dbp4-GFP and αSyn-mCherry. Multiple fluorescent foci are visible that spread in the nucleoplasm (Fig 5E and 5H) indicating nucleolar stress [19]. In order to assess, whether the Dbp4-GFP foci are truly nucleolar fragments, colocalization experiments were performed with the nucleolar marker Nop1-RFP, expressed from a single copy plasmid (Fig 5F). Dbp4 signal colocalized with Nop1 that confirmed the nucleolar localization of Dbp4 and indicated that the Dbp4 foci are nucleolar fragments.

**Fig 6:**
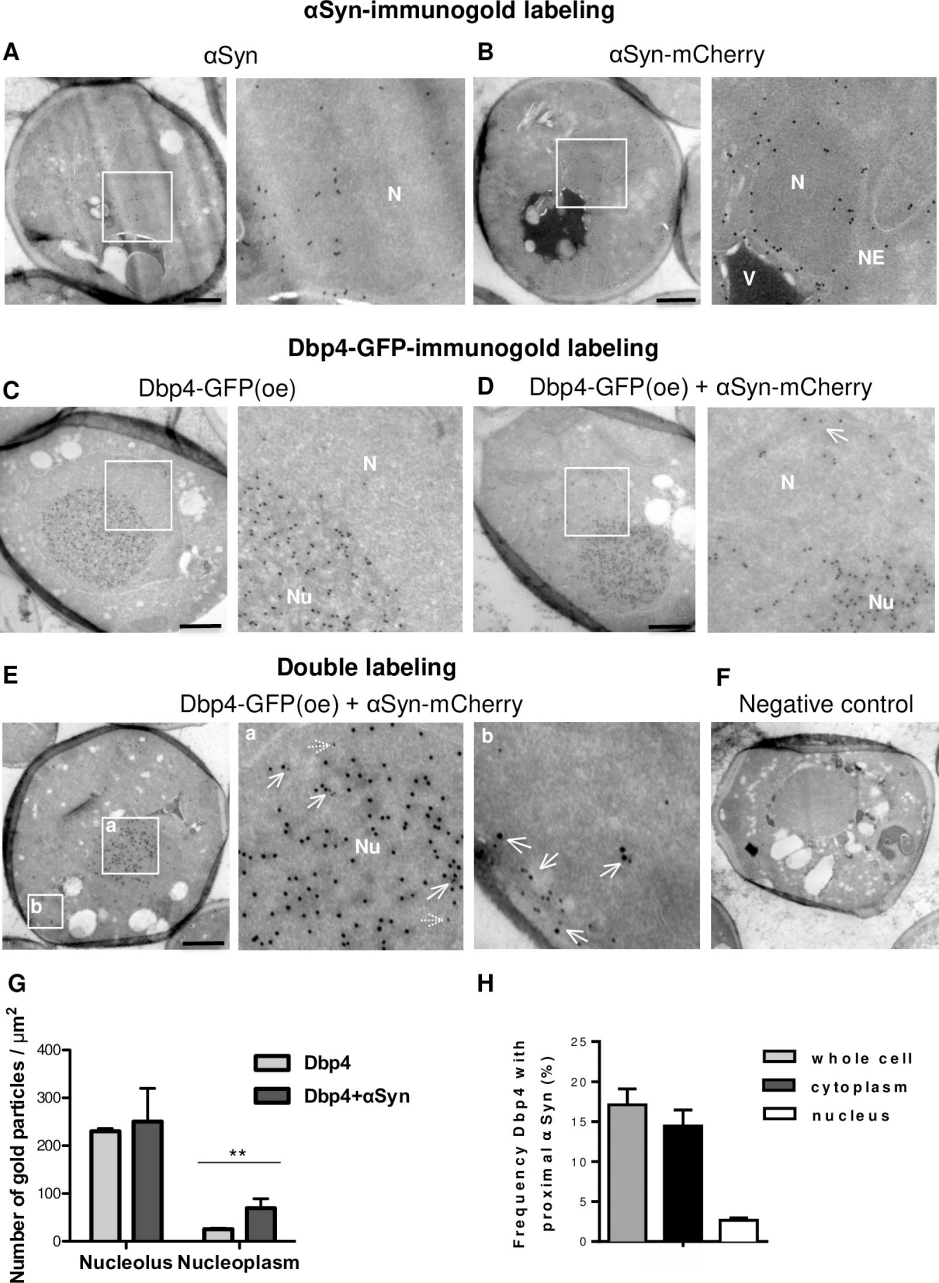
αSyn sequesters Dbp4 outside the nucleolus. Representative electron micrographs showing immunogold labeling of αSyn, αSyn-mCherry and Dbp4-GFP in yeast cells. Right: micrographs with higher magnifications of indicated areas marked by white boxes. **(A)** αSyn-positive gold particles are in the nucleus. Nucleolus is not visible. **(B)** αSyn-mCherry shows similar subcellular localization as the untagged protein. **(C)** Overexpression (oe) of Dbp4-GFP and immunogold labeling results in nucleoli with high densities of gold particles, but only a few gold particles scattered in nucleoplasm. **(D)** Co-expression of Dbp4-GFP and αSyn-mCherry. Gold particles are distributed in nucleoplasm with higher density. **(E)** Double immuno-labeling of αSyn and Dbp4-GFP. 15 nm gold particles were used for GFP-labeling and 10 nm gold particles for αSyn labeling. Dashed arrows indicate single αSyn-gold particles; tick arrows indicate colocalization of αSyn and Dbp4-gold particles. N: nucleus; NE: nuclear envelope; Nu: nucleolus; V: vacuole. Scale bar = 500 nm. **(F)** Negative control for immuno-labeling. Yeast cells were processed as in (E), however the primary antibodies were omitted. **(G)** Distribution of Dbp4-GFP-gold particles in the nucleus. Expression of αSyn significantly increases the localization of Dbp4-GFP outside the nucleolus. Significance of differences was calculated with t-test (**p < 0.01, n = 37). **(H)** Frequency of co-localization of αSyn and Dbp4. Dbp4-gold particles that localize in a proximity of αSyn-gold particles per cell, in the cytoplasmic or in the nuclear region were counted and referred to the total number of Dbp4-gold particles per cell.

Immunoelectron microscopy (immunoEM) was performed with ultrathin cryosections and antibodies directed against αSyn or GFP to further evaluate the consequences of αSyn expression on Dbp4 localization at the nanometer level. Cells expressing Dbp4-GFP and αSyn-mCherry were utilized for consistency with the fluorescence microscopy studies. Non-tagged αSyn used as control revealed a similar immunogold labeling pattern as αSyn-mCherry, confirming the nuclear localization of the protein (Fig 6A and 6B). Dbp4-GFP expressed from its native promoter was below the detection limit. Overexpression of *DBP4* and immunolabeling in the absence of αSyn-mCherry demonstrated large nucleoli with well-defined boundaries (Fig 6C). Co-expression with αSyn-mCherry resulted in less defined boundaries and spreading of the gold particles in the nucleoplasm outside of the dense nucleolar region (Fig 6D and 6G). In addition, double-label immunoEM demonstrated co-localization of Dbp4-GFP and αSyn-mCherry in the nucleolus, as well as in the cytoplasm near the plasma membrane (Fig 6E). The frequency of co-localization of αSyn and Dbp4 was evaluated from EM immunostaining images. The Dbp4-GFP-gold particles that localized in a proximity of αSyn-gold particles in the whole cell, in the cytoplasm or in the nucleus were counted and referred to the total number of Dbp4-GFP-gold participles per cell (Fig 6H). About 14.5% of the total immunostained Dbp4 co-localized with αSyn in the cytoplasm, and 2.66% of the Dbp4-gold particles were localized in a proximity of αSyn-gold particles in the nucleus. These results are consistent with the fluorescence microscopy and BiFC studies and verify that αSyn co-localizes with Dbp4 and causes its mislocalization outside of the nucleolar region.

### αSyn interacts with and sequesters DDX10 into cytoplasmic inclusions in human cells

**Fig 7:**
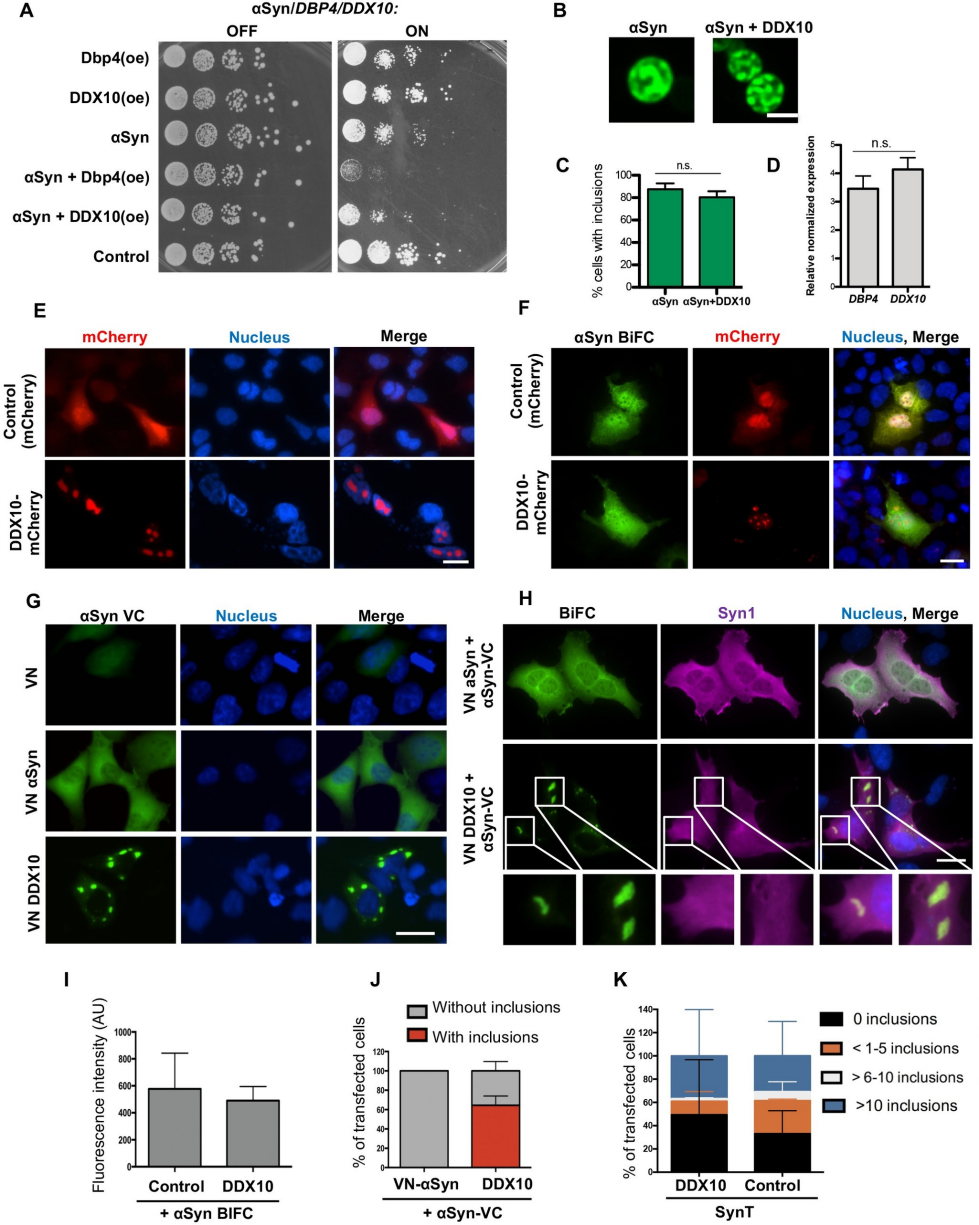
αSyn interacts with human DDX10 (ortholog of yeast Dbp4) in human cells. **(A)** *DDX10* overexpression enhances αSyn toxicity in yeast. Growth assay of yeast cells expressing *GAL1*-driven αSyn-GFP, *DDX10* or *DBP4* from 2μ plasmids with empty vector as control. Yeast cells were spotted in 10-fold dilutions on selection plates containing glucose (*GAL1—*OFF) or galactose (*GAL1—*ON). **(B)** Fluorescence microscopy of yeast cells, expressing αSyn-GFP in presence or absence of *DDX10* overexpression. Cells were imaged 6 h after induction of protein expression in galactose-containing medium. Scale bar = 5 μm. **(C)** Quantification of the percentage of cells displaying αSyn-GFP inclusions. Significance of differences was calculated with t-test (n.s., n = 3). **(D)** Relative abundance of *DBP4* and *DDX10* mRNA in cells from (B), determined by qRT-PCR. Significance of differences was calculated with t-test (n = 4). **(E)** Fluorescence microscopy of Human Embryonic Kidney cells (HEK), expressing mCherry (control) or DDX10-mCherry. DDX10-mCherry localizes exclusively in the nucleoli. **(F)** HEK cells were transfected with BiFC constructs encoding αSyn tagged with either N-terminal (VN) or C-terminal (VC) half of Venus under the control of the CMV promoter. Representative pictures of αSyn oligomerization upon overexpression of DDX10-mCherry. Expression of mCherry was used as a control. **(G)** DDX10 interacts with αSyn outside the nucleus. Fluorescence microscopy of cells transfected with VN-αSyn and αSyn-VC or VN-DDX10 and αSyn-VC. VN served as a negative control. BiFC reconstitution of Venus fluorescence reveals that αSyn-DDX10 interactions lead to cytosolic accumulation of both proteins. **(H)** Intracellular distribution of αSyn. Fluorescence microscopy of cells transfected with VN-αSyn and αSyn-VC or VN-DDX10 and αSyn-VC (BiFC) and stained with antibody against αSyn (Syn1). Nuclei are stained with Hoechst dye. Scale bar: 25 μm. Lower panels: higher magnifications of the indicated areas marked by white boxes. **(I)** Oligomerization of αSyn. Mean fluorescence intensity of cells from (B), expressing αSyn BiFC constructs with or without DDX10-mCherry. **(J)** Quantification of cells, displaying fluorescent inclusions upon expression of VN-DDX10 and αSyn-VC. **(K)** DDX10 does not alter the inclusion formation of αSyn. HEK cells were co-transfected with constructs expressing SynT, synphilin-1 and DDX10 or pcDNA (control). Quantification of the percentage of cells displaying αSyn inclusions 48 h after transfection. Cells were classified into 4 groups according to the number of inclusions per cell.

The ribosome biogenesis is a highly conserved essential pathway in eukaryotes [42]. *DDX10* as human ortholog of *DBP4*, was found in the human small subunit processome and is likely to fulfil similar functions as Dbp4 [39,41,43]. We tested whether human DDX10 has the same impact on αSyn-induced toxicity in yeast cells as Dbp4. Overexpression of *DDX10* enhanced the growth inhibition of αSyn-expressing cells similar to *DBP4*, also without significantly affecting αSyn inclusion formation (Fig 7A–7C). The mRNA levels upon overexpression of the two genes in yeast were similar (Fig 7D). This corroborates the results for Dbp4 and suggests DDX10 as a human αSyn toxicity enhancer.

Human Embryonic Kidney 293 (HEK) cells were used to validate our findings in the context of a human cell line. Expression of DDX10-mCherry in HEK cells exhibited a typical nucleolar morphology, revealing multiple nucleoli per nucleus (Fig 7E). αSyn oligomerization visualized by BiFC [8] was not altered by *DDX10* overexpression (Fig 7F and 7I). BiFC assays of human DDX10 with αSyn fused to the complementary N- and C-terminal fragments of the Venus reporter transfected in HEK cells revealed that the two proteins interact and form large cytoplasmic inclusions in ~60% of the cells (Fig 7G and 7J). Cells expressing the BiFC constructs were immunostained with an anti-αSyn antibody to confirm the subcellular localization of the protein, that was distributed throughout the cytoplasm and nucleus [9] (Fig 7H). The Syn1 signal revealed similar diffuse staining in the cytoplasmic inclusions and did not accumulate there, suggesting that DDX10 does not influence αSyn localization.

The interaction of αSyn with Dbp4 or DDX10 was tested using purified proteins and pull-down *in vitro* assays. His6-tagged Dbp4 or DDX10 were incubated alone or in presence of purified αSyn (no tag) and bound to Ni-NTA beads. αSyn was selectively eluted together with the two proteins, demonstrating a direct interaction between αSyn and Dbp4/DDX10 (S9 Fig).

αSyn aggregation can be induced by co-expressing C-terminally modified αSyn (SynT) and synphilin-1, an αSyn-interacting protein that is also present in LBs [8]. Similar to the finding from yeast cells, we found that overexpression of DDX10-mCherry did not change the percentage of cells with αSyn inclusions in this established human aggregation model (Fig 7K).

These results corroborate αSyn interaction with human DDX10 similar to yeast Dbp4. Elevated levels of either protein do not increase αSyn inclusion formation but instead, αSyn sequesters DDX10/Dpb4 outside of the nucleus. In contrast to yeast, where only a faint cytoplasmic BiFC staining is observed, DDX10 and αSyn form big cytoplasmic inclusions in human cells.

### Dbp4/DDX10 induce oligomerization and reduce the fibrilization of αSyn

**Fig 8:**
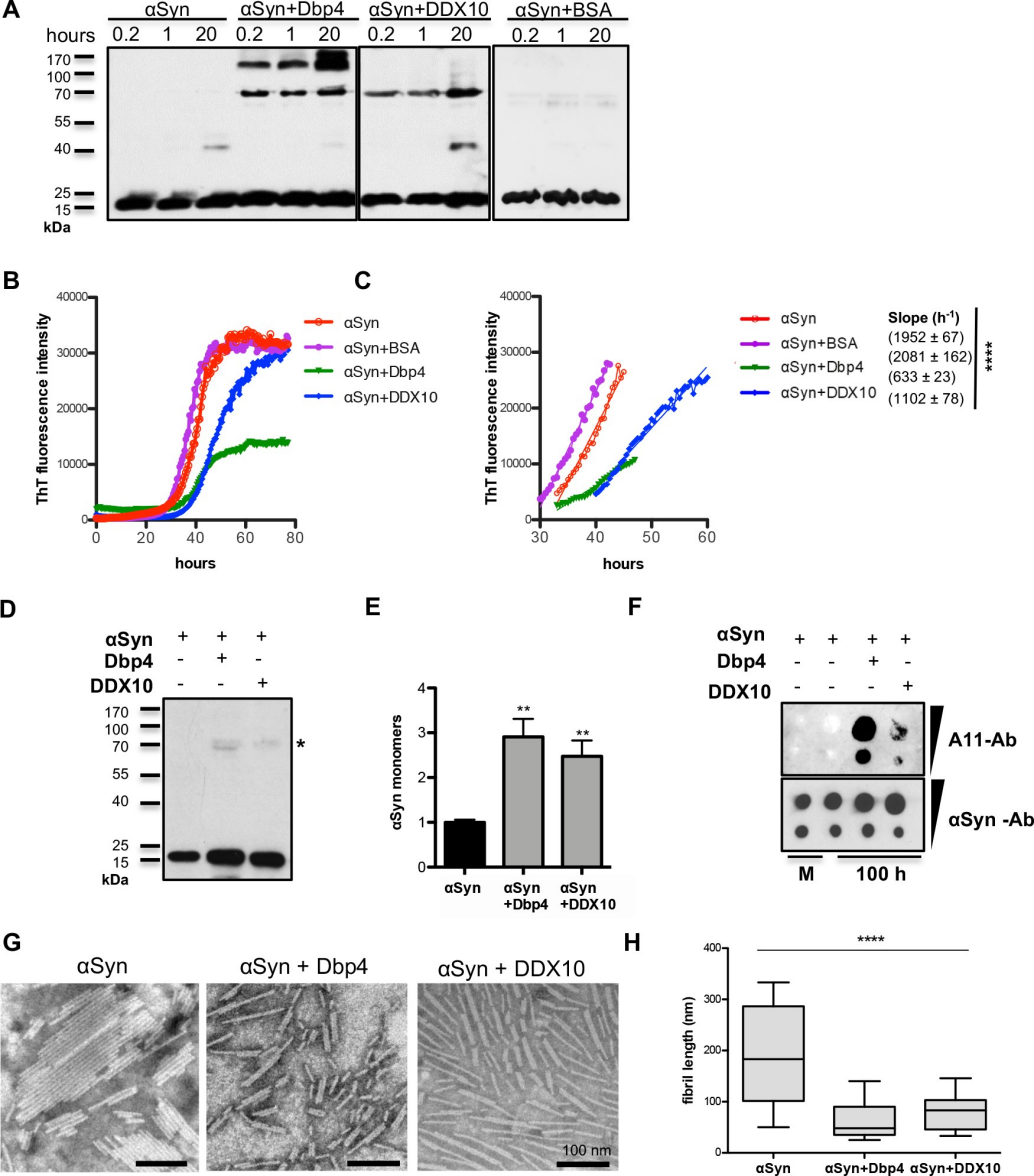
Dbp4 and DDX10 induce oligomerization and reduce the fibrilization of αSyn. **(A)** Immunoblotting of purified αSyn incubated either alone or with Dbp4, DDX10 or BSA purified proteins at molar ratio 2:1 (C_αSyn_ = 10 μM) using αSyn antibody. Equal aliquots were taken after 10 min, 1 h and 20 h. **(B)** Aggregation kinetics of αSyn in the presence or absence of Dbp4, DDX10 or BSA as a control, monitored by ThT fluorescence emission. The ThT signal was recorded every 2 minutes for 80 h. Representative curves from three independent experiments are presented. (**C)** Slope of the curves in exponential phase. Significance of differences was calculated with One-way ANOVA (****p < 0.0001). **(D)** Immunoblotting of equal amounts of samples from end point products from (B) using αSyn antibody. **(E)** Quantification of the band density representing the amount of monomeric αSyn of samples from (D). The signal intensity was normalized to αSyn alone (n = 3). **(F)** Dot-blot analysis of samples from end point products from (B). 10 μl and 2 μl from each sample were applied on a membrane and probed with A11 anti-oligomer antibody. The membrane was stripped and probed with αSyn antibody that recognizes all protein species as a control for adsorbed protein onto the membrane. M: monomeric αSyn. **(G)** TEM images obtained with end point products of the kinetic run (B) reveal formation of mature amyloid fibrils. **(H)** Measurements of the mean length of αSyn fibrils in absence or presence of Dbp4 or DDX10. Significance of differences was calculated with One-way ANOVA (****p < 0,0001, n = 150).

As *DBP4* overexpression increased the fraction of oligomers in yeast nuclei (Fig 3C), we assessed *in vitro* whether Dbp4 or DDX10 have a direct impact on αSyn oligomerization or fibrilization. αSyn was incubated alone or in presence of either purified Dbp4 or DDX10 proteins and the formation of SDS-stable oligomers at 4°C was analysed using immunoblotting with αSyn-antibody (Fig 8A). αSyn alone was unable to form oligomers in this time frame and only a faint band with mobility of ~40 kDa corresponding to αSyn dimers was detected after 20 h incubation. Addition of Dbp4 or DDX10, but not of bovine serum albumin (BSA) as control, induced αSyn oligomer formation already after 10 min with mobilities of ~70 kDa (Dbp4 and DDX10) or ~100 kDa (Dbp4). The 70 kDa oligomers correspond to the identified oligomeric species in yeast nuclei and those co-sedimenting with pre-ribosomes (Fig 3C and 3H).

The kinetics of αSyn amyloid formation was followed by continuously monitoring the changes in thioflavin T (ThT) fluorescence over time in the absence or presence of Dbp4 or DDX10 (Fig 8B). αSyn aggregation followed a sigmoidal curve, reflecting a nucleation-dependent growth mechanism. The curve generated in the presence of Dbp4 reached a plateau at 2.5-fold lower fluorescence intensity than αSyn alone or in the presence of DDX10. Following a lag phase of approximately 30 h, Dbp4 and DDX10 significantly reduced the aggregation rate, indicated by the slope of the curves in exponential phase (Fig 8C). Western blot analysis was performed with equal amounts of samples collected from end points of the aggregation reactions to quantify the amount of aggregated protein (Fig 8D). The amount of αSyn monomers in the samples co-incubated with either Dbp4 or DDX10 was higher when compared to αSyn alone that demonstrates less efficient incorporation of αSyn monomers into fibrils (Fig 8E). Furthermore, high-molecular weight SDS-stable oligomers were detected in the samples, co-incubated with Dbp4 or DDX10. For characterization of the oligomeric species under non-denaturing conditions, dot-blot analysis was performed with anti-oligomer A11 antibody that binds oligomeric αSyn, but not monomeric or fibrillar forms (Fig 8F). Oligomers were not detected in αSyn alone after prolonged incubation, similar to the control monomeric αSyn. However, oligomers were abundant in the presence of Dbp4 and were also observed at lower levels in the presence of DDX10.

The morphological features of the samples at the end point of the aggregation reactions were imaged using transition electron microscopy (TEM) (Fig 8G). The size and morphology of the fibrils formed in the presence and absence of Dbp4/DDX10 clearly differed. αSyn alone formed long amyloid fibrils, whereas the fibrils formed in the presence of Dbp4 or DDX10 were considerably shorter (Fig 8H).

These data suggest complex kinetics of αSyn fibrilization, where Dbp4/DDX10 stimulate the earliest stages of oligomerization and later on inhibit αSyn fibrilization. Dbp4/DDX10 stabilized a fraction of oligomeric species that were not incorporated into larger fibrils after prolonged incubation and inhibited the fibrilization, consistent with our observations of αSyn fibril morphology.

## Discussion

We performed pioneering genome-wide screens of yeast essential genes to identify proteins that modulate αSyn toxicity. Overexpression of αSyn in various eukaryotic systems, including yeast, has been used for identification of cellular processes and genes, associated with PD [20], however systematic studies of the essential cellular pathways were still lacking. Our approach revealed genetic interactions between αSyn and proteins encoded by essential genes for which there was previously no known relationship. In both yTHC and DAmP screens, the identified gene hits were clustered in the central proteostasis pathways of protein synthesis and ubiquitin-dependent protein degradation, however, only seven hits overlapped between the two screens (S2 Table). Individual genes can escape detection during high throughput screening or might reveal variable cellular responses towards αSyn expression in different strains. The differences of DAmP and yTHC library responses towards αSyn expression are probably due to the different level and nature of downregulation of the essential genes in the two libraries. DAmP alleles exhibit a mild effect on cellular fitness as only a fraction of them compromised gene function enough to reveal >5% fitness defect [44].

**Fig 9:**
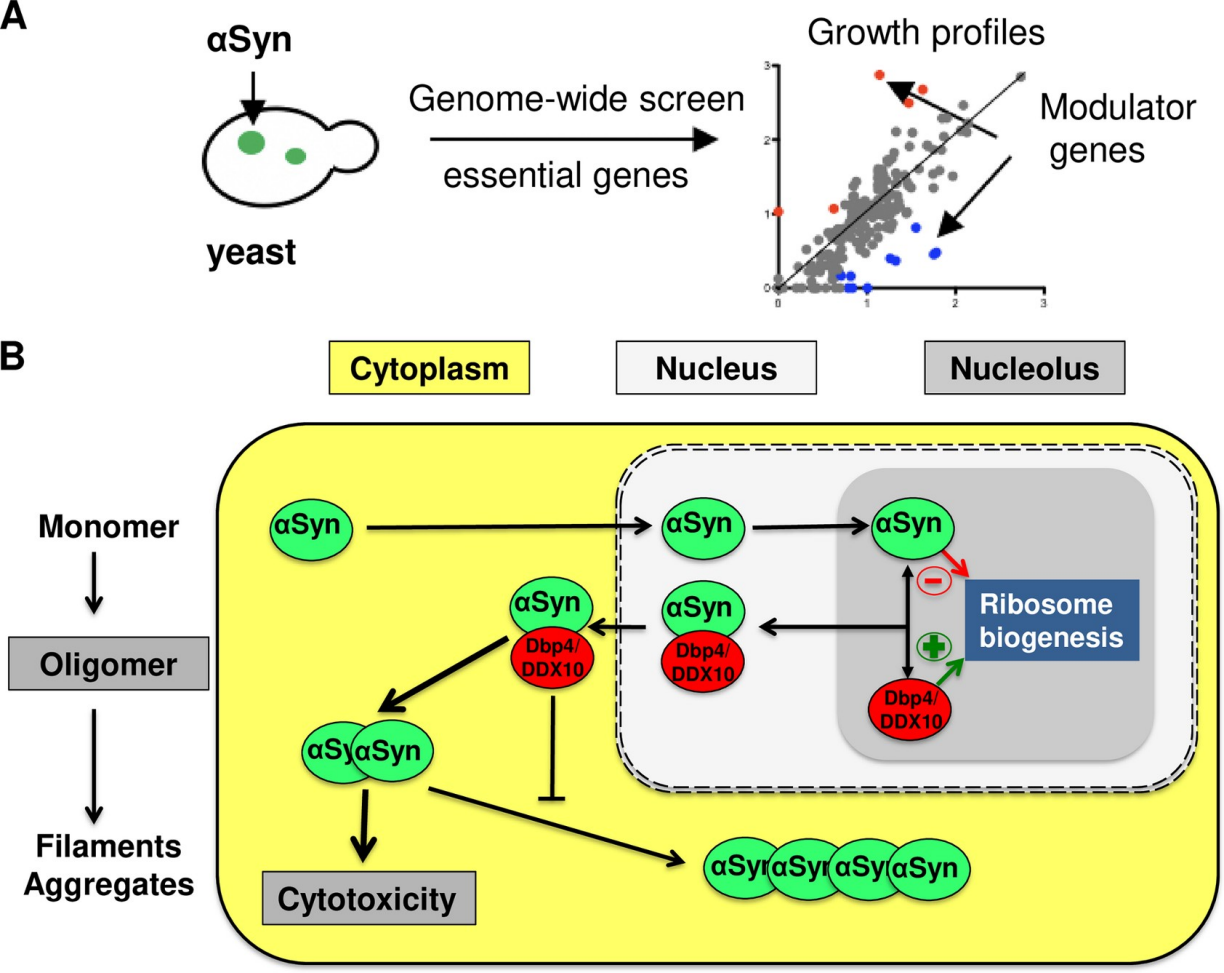
αSyn affects ribosome biogenesis and sequesters Dbp4/DDX10 out of the nucleolus. **(A)** Genome-wide screens with conditional alleles of yeast essential genes resulted in identification of 98 modulators of αSyn toxicity. Significant portion of the identified modifiers are nucleolar proteins. **(B)** αSyn affects ribosome biogenesis and causes accumulation of pre-rRNA transcripts in yeast and in human cells. The DEAD-box RNA helicase Dbp4/DDX10 links nucleolar activity and αSyn-induced toxicity. αSyn interacts with Dbp4/DDX10, sequesters the proteins out of the nucleus and especially in human cells into cytoplasmic inclusions. αSyn-Dbp4/DDX10 interactions enhance cytotoxicity by promoting αSyn oligomerization and reducing αSyn fibril formation.

A significant portion of the identified nuclear modulators are localized in the nucleolus, which suggests that nucleolar processes contribute to the toxicity effect of αSyn. We identified the nucleolar DEAD-box helicase Dbp4/DDX10 as a strong enhancer of αSyn-induced toxicity. Dbp4/DDX10 interacted with αSyn, promoted its oligomerization and sequestered Dbp4/DDX10 outside of the nucleus. Our results reveal synergistic enhancement of αSyn toxicity due to Dbp4/DDX10 sequestration and Dbp4/DDX10-induced αSyn oligomer formation (Fig 9).

Neurodegenerative diseases can be associated with nucleolar stress caused by impaired ribosome assembly or altered nuclear integrity but the molecular basis of these observations and the regulators that contribute to the nucleolar stress in PD are still not understood [10,13,15,45]. 18S rRNA levels decrease with aging in human and mice and altered nucleolar function and morphology have been described in dopaminergic neurons of PD brains [15,46]. For still unclear reasons, inhibition of rRNA synthesis can be both neuroprotective and neurotoxic [45]. One possible explanation is that the nuclear subpopulation of αSyn affects the subcellular localization of components of the ribosome biogenesis machinery.

Here, various genes encoding ribosome biogenesis factors were identified in both yTHC and DAmP screens, which further supports the link between the nucleolar activity and αSyn toxicity. Three genes *(DBP4*, *RCL1*, *NOP4)* were further characterized as regulators of αSyn-mediated toxicity. While Dbp4 and Rcl1 are components of the SSU processome and required for 18S rRNA biogenesis, Nop4 is involved in the processing to generate the mature 25S rRNA during assembly of the large ribosomal subunit [47]. We demonstrate for the first time that αSyn is localized in the nucleolus in yeast. This supports recent observations in a novel mouse digenic model of PD [13]. αSyn interacted with Dbp4 and DDX10, components of the yeast and human SSU processome respectively. In human cells and yeast expressing Dbp4 at its endogenous level, expression of αSyn leads to pre-rRNA accumulation. The mechanistic basis of the observed alterations in pre-rRNA levels observed in cells expressing αSyn remains unclear as well as how this is linked to Dbp4/DDX10. It is possible that the changes in pre-rRNA levels could arise due to enhanced rRNA transcription or alterations in the kinetics of pre-rRNA processing steps. Upon co-overexpression, the two proteins interact with each other and are sequestered out of the nucleolus. Lack of Dbp4 impairs various early pre-rRNA processing steps manifesting in accumulation of the 35S pre-rRNA and lack of the 20S pre-rRNA. This phenotype is not mirrored upon expression of αSyn implying that the observed pre-rRNA processing defects do not simply arise due to withdrawal of essential Dbp4 from the nucleolus. The results rather imply that αSyn expression affects nucleolar processes in general and that this in turn affects pre-rRNA transcription or processing. This notion is supported by the observation that the catalytic activity of Dbp4 is required for its role in ribosome assembly, whereas introduction of mutations impairing ATP binding and hydrolysis does not affect the influence of Dbp4 on αSyn-induced toxicity. It is likely that the effect of Dbp4 on αSyn toxicity arises due to an additional function of the protein, for example, in regulating αSyn oligomerization (see below).

DDX10 emerged as novel target against αSyn-induced damage, because we demonstrated that αSyn-induced toxicity correlates with elevated expression levels of *DBP4/DDX10*. An elevated level of DDX10 is linked to diseases such as cancer or neurodegeneration. *DDX10* is significantly overexpressed in osteosarcoma cancer patients [48]. *DDX10* was also reported to be up-regulated in different brain regions of Atypical Frontotemporal Lobar Degeneration [49], however the molecular basis of this effect remains unknown. Importantly, αSyn interacted with Dbp4 and DDX10 and sequestered the nucleolar proteins into the cytoplasm in yeast and in human cells. This effect was very prominent in human cells, where DDX10 and αSyn formed big cytoplasmic inclusions, and less prominent in yeast, where a slight BiFC complementation signal was observed in the cytoplasm near the plasma membrane that resemble αSyn inclusions. Although different approaches clearly demonstrated interaction of αSyn with Dbp4 in the cytoplasm of yeast cells, it is not obvious whether Dbp4 and αSyn form inclusions, similar to human cells.

Until now, only a few proteins have been described to promote the formation of αSyn cytosolic inclusions in cellular models [50,51]. Sequestration of cellular-interacting functional proteins into protein aggregates leads to cytotoxicity and neurodegeneration [52]. Future studies remain to determine whether αSyn binds Dbp4/DDX10 in the cytoplasm *en route* to its destination to the nucleolus and traps it in cytoplasmic aggregates, thus preventing its proper localization and function. Alternatively, αSyn may hijack Dbp4/DDX10 in the nucleolus and sequester it to the cytoplasmic inclusion. αSyn expression resulted in release of nucleolar Dbp4 protein into the nucleoplasm and caused nucleolar disruption. Disruption of nucleolar integrity has been observed in human post mortem samples from patients with PD and is connected to increased oxidative stress and mitochondrial dysfunction [15].

We show that Dbp4 and DDX10 promote oligomerization of αSyn *in vitro* and lead to the formation of shorter fibrils. This was accompanied by inhibition of αSyn fibrilization. Dbp4/DDX10 stabilized a fraction of oligomeric species that were not incorporated into larger fibrils. These oligomers probably have a different conformation and/or toxicity and their stabilization and accumulation might be the major contributor to the toxicity enhancement effect of Dbp4/DDX10. The stabilization of the oligomeric species is probably mediated by binding of the proteins to the oligomers. This corroborates the direct correlation between the expression level of *DBP4/DDX10* and αSyn-induced toxicity, as elevated protein concentration would increase the level of toxic oligomers. αSyn oligomers exist in different conformations and structures, and might exert various harmful effects [53]. Elevated levels of Dbp4 increased the abundance of αSyn 70kDa oligomeric species in the nucleus. Our data are consistent with previous findings, showing the existence of an αSyn oligomeric species in isolated nuclei from cells of the frontal cortex area of PD patients [10]. Thus, Dbp4/DDX10 might induce formation of compartment-specific conformers with different propensities for cellular toxicity. This highlights DDX10 as a promising target for a therapeutic strategy based on drug-induced regulation of target expression.

## Materials and methods

Yeast strains, plasmids and other resources and tools, used in the study are listed in S6 Table.

### Yeast transformation and growth conditions

*S*. *cerevisiae* strains were used for transformations performed by standard lithium acetate protocol [54]. Yeast strains were grown at 30°C in non-selective YEPD (Yeast Extract—Peptone—Dextrose) or synthetic complete dropout (SC) medium [55] lacking the relevant amino acids for selection, supplemented with 2% glucose, 2% raffinose or 2% galactose. For down-regulation of the essential genes from the yeast Tet-Promoters Hughes Collection (yTHC), the medium was supplemented with 10 μg/ml doxycycline. Expression of *GAL1*-αSyn was induced by shifting overnight cultures from 2% raffinose to 2% galactose-containing SC selection medium at *A*_*600*_ = 0.1 for non-toxic strains or *A*_*600*_ = 0.3 for toxic strains.

### Cloning of recombinant DNA

Yeast plasmids were constructed using GENEART Seamless cloning and assembly kit (ThermoFisher) according to the manufacture instructions. The Dbp4 mutant constructs were generated by site-directed mutagenesis using QuikChange II Site-Directed Mutagenesis Kit (Agilent Technologies). All constructs were verified by DNA sequencing.

### Synthetic Genetic Array (SGA) analysis

Synthetic genetic array (SGA) technology [33,56] was employed for analysis of the synthetic genetic interactions among the conditional alleles of essential genes from two collections and the *SNCA* gene encoding human αSyn. The yTHC collection contains 844 strains where the expression of essential yeast genes is regulated by doxycycline [31]. The second collection comprises the decreased abundance by mRNA perturbation (DAmP) alleles, which can lead to reduced transcript levels [32,57].

First, a query strain RH3795 was constructed that harbored two genomically integrated copies of *GAL1*-driven *SNCA* gene, fused to GFP. Yeast strain Y7092 was transformed with the linearized integrative plasmid pME5038 using standard lithium acetate procedure [54], conferring prototrophy to leucine. Since the auxotrophic marker mutation in the Y7092 is a null deletion, *TRP1* was cloned into the vector in PdiI restriction site, allowing tandem integration into the *TRP1* locus of Y7092 strain via homologous recombination. The number of integrated copies was verified by Southern blotting as previously described [22].

The query strain was crossed to an ordered array of conditional mutants as described below using Singer ROTOR HDA bench robot (Singer Instruments, UK) with manufacturer’s software and disposable plastic replicator pads. Because ammonium sulphate impedes the function of G418, synthetic medium containing this antibiotic was made with monosodium glutamic acid as a nitrogen source (20 g/l agar, 1.7 g/l yeast nitrogen base without ammonium sulphate and amino acids, 1 g/l monosodium glutamic acid, 2 g/l amino acid drop-out mix lacking the marker amino acids). Where indicated, the media was supplemented with 50 mg/l canavanine, 50 mg/l thyalisine or 200 mg/l geneticin (G418).

The query strain RH3795 was crossed with an array of yTHC strains of the opposite mating type *MAT*a, carrying a KanR marker conferring resistance to G418 and Ura-marker. To generate a source of newly grown cells for mating, the query strain was grown overnight in 5 ml of selection medium (SC-Leu), and then plated onto rich (YEPD) plates. The yTHC library collection was replicated onto fresh SC-Ura + G418 plates in 96-format density. The plates were incubated at 30°C for two days. The yTHC strains were mated with the query strain by first pinning the 96-format query strain on fresh YEPD plates, and then pinning the yTHC on top of the query strain. The plates were incubated at room temperature (RT) for one day. The resulting *MAT***a/**α zygotes were pinned onto solid medium that selects for growth of diploid cells (SC-Leu-Ura + G418) and the plates were incubated at 30°C for two days. The diploids were transferred to a medium with reduced level of carbon and nitrogen (2% agar, 1% potassium acetate, 0.1% yeast extracts, 0.05% glucose, 50 mg/l G418, supplemented with uracil, histidine, lysine and leucine) to induce sporulation and formation of haploid meiotic spore progeny and the plates were incubated for 7 days at 22°C. For selection of *MAT*a haploid meiotic progeny, the spores were pinned on Ha selection medium (SC-His-Arg-Lys + canavanine + thialysine) and incubated at 30°C for two days. The *MAT*a meiotic progeny was pinned onto plates with the same medium for a second round of selection and grown for one day at 30°C. For selection of query (αSyn) allele, the cells were pinned onto Hq medium (SC-Leu-His-Arg-Lys + canavanine + thyalisine) and grown for two days at 30°C. To select for meiotic progeny that carries the conditional alleles derived from the parental yTHC strains, *MAT***a** haploids were pinned onto Hd plates (SC-Ura-Leu-His-Arg-Lys + canavanine + thyalisine + G418) and the plates were incubated for two days at 30°C.

Two independent SGA screens with the yTHC collection were carried out. The ability of each conditional allele to worsen or improve the growth deficiency resulting from accumulation of αSyn was monitored. The cells from the final selection were spotted in quadruplicates in 384-density format on SC-Ura-Leu selection plates supplemented with: (i) 2% glucose (control); (ii) 2% galactose (induction of *GAL1* promoter); (iii) 2% glucose + 10 μg/ml doxycycline (downregulation of *Tet*-promoter); (iv) 2% galactose + 10 μg/ml doxycycline (downregulation of *Tet*-promoter and induction of *GAL1* promoter). The growth rates of the final double-mutant haploid strains were monitored visually. A visible colony growth was observed for all strains even by downregulation of the *Tet*-promoter due to the nature of the robotic arraying system used for yeast high throughput screens that deposits significant number of cells on the test plates and because of the delay of several hours in the onset of growth cessation in the *Tet*-promoter strains. The potential positive hits from the two rounds of screening, showing smaller or bigger colony size on plates (iv) (*Tet*-OFF + *GAL1*-ON) in comparison with plates (ii) (*Tet*-ON + *GAL1*-ON) or plates (iii) (*Tet*-OFF + *GAL1*-OFF) were combined and 92 strains were used for a new SGA screen. The genetic interactions from the third screen were scored by measuring the area of individual colonies using Balony software [58]. The program uses an algorithm for colony area normalization that corrects for multiple possible biases. First, colony areas are normalized to the median of the plate that removes inconsistences due to variable image size or differences in plate thickness, followed by row/column and spatial corrections, correcting for edge effects due to more access of nutrients or correlations in colony sizes in a region of the plate. Finally, a competition correction is performed, necessary when a colony has a number of slow-growing colonies surrounding it [58]. The colony areas were paired by the Balony software to the control plate (*Tet*-ON, αSyn-OFF) that takes into account differences in growth under normal conditions and yields relative colony size for each colony on the experimental plates relative to the control plate. Since the endogenous promoter of each essential gene is replaced with the *Tet*-promoter, a number of mutant alleles showed abnormal growth phenotypes even in the absence of doxycycline due to up- or down-regulation of their expression [31]. Approximately 20% of all clones from the collection show smaller colonies even in absence of doxycycline, suggesting that these genes are very sensitive to changes of the promoter activity [31]. The response to doxycycline varied among the different strains, presumably due to the different response of the cells to transcript depletion. Fifty putative interactions generated from SGA analysis were analysed individually by random spore analysis as described below. Finally, the hits were verified by spotting assay. These two assays are more sensitive and resulted in stronger synthetic sick/lethal phenotypes. Twenty-one strains showed genetic interactions between αSyn and *Tet*-alleles of essential genes, sixteen strains showed dox-sick/lethal phenotypes, seven were galactose-sick/lethal and six did not show a synthetic growth phenotype.

The screen with the DAmP collection was performed using the SGA technology as described above with modifications. Control strain RH3796 was constructed that harbors empty vector conferring prototrophy to leucine by transformation of yeast strain Y7092 with the empty integrative vector pME5037 using standard lithium acetate procedure [54]. The DAmP collection consisting of 842 *MAT*a haploid strains was crossed with the query strain RH3795 or with the control strain RH3796 using the same steps and selection media as described above, however supplementing all media with uracil. The final haploid double mutant strains were used for growth rate measurements. The cells were spotted on SC-Leu + G418 selection plates, supplemented with 2% glucose or 2% galactose. Cells crossed with the empty vector strain RH3796 served as control. Colony area was measured from digital images of the plates using Balony software [58]. The colony areas were normalized by the software as described above and a growth fit factor was calculated as ratio of *area αSyn-strain / area control strain* on galactose plates. Four independent SGA screens were performed. A growth defect was considered significant by the following criteria: mean growth fit factor < 0.85; standard deviation < 0.05; n = 4.

### Random spore analysis

Random spore analysis (RSA) was performed as described [33] with modifications as follows. A small amount of spores, saved from the sporulation step were resuspended in 1 ml sterile water and plated on the following media: 1) SC-Ura/His/Arg/Lys + canavanine + thyalysine + glucose (selection for all haploid spore products of *Mat*a); 2) SC-Ura/Leu/His/Arg/Lys + canavanine + thyalysine + G418 + glucose (selection for haploid *Tet*-allele and αSyn-allele); 3) SC-Ura/Leu/His/Arg/Lys + canavanine + thyalysine + G418 + doxycycline + glucose (selection for haploid *Tet*-allele and αSyn-allele, shut-off of *Tet*-promoter); 4) SC-Ura/Leu/His/Arg/Lys + canavanine + thyalysine + G418 + galactose (selection for haploid *Tet*-allele and αSyn-allele, shut-on of *GAL1*-promoter); 5) SC-Ura/Leu/His/Arg/Lys + canavanine + thyalysine + G418 + doxycycline + galactose (selection for haploid *Tet*-allele and αSyn-allele, shut-on of *GAL1*-promoter; shut-off of *Tet*-promoter). The plates were incubated at 30°C for 3 days and scored by comparison of the cell growth on the five plates. The strains that grew slowly on doxycycline were scored only after colonies appeared on the plates.

### Spotting assays

To investigate growth on solid medium, yeast cells were pre-grown in selective SC medium containing 2% raffinose lacking the corresponding marker. After normalizing the cells to equal densities (*A*_*600*_ = 0.1), 10-fold dilution series were prepared and spotted in a volume of 10 μl on SC-selection agar plates supplemented with either 2% glucose or 2% galactose. Where indicated, the plates were supplemented with 10 μg/ml doxycycline. The growth was documented after incubation for three to four days at 30°C.

### RNA preparation and quantitative real-time PCR

Total RNA was isolated from yeast cells that were grown for 6 h in SC selection medium supplemented with 2% galactose in presence or absence of 10 μg/ml doxycycline using the High Pure RNA Isolation Kit (Roche Diagnostics GmbH, Germany). cDNA synthesis was performed using 0.8 μg RNA and the QuantiTect Reverse Transcription Kit (Qiagen, Germany) according to the manufacturer’s instructions. 20 ng of cDNA was used as a template for quantitative real-time PCR experiments and amplification was performed using CFX Connect Real-Time System (BioRad Laboratories, Germany). Three independent biological replicates and four technical replicates each were performed for the genes of interests or *H2A* as a reference and analyzed with the CFX Manager Maestro software (Bio-Rad Laboratories, Germany).

### RNA preparation and northern blotting

Total RNA was extracted from yeast cells as in [59]. Exponentially growing cells were harvested and resuspended in equal volumes of GTC mix (2 M guanidine thiocyanate, 25 mM Tris/HCl pH 8.0, 5 mM EDTA pH 8.0, 1% (v/v) N-lauroylsarcosine, 150 mM ß-mercaptoethanol) and acidic phenol. Cells were lysed by mechanical disruption with glass beads, and samples were incubated at 65°C for 5 min before addition of chloroform and vortexing. Samples were centrifuged at 20000 x g for 5 min, the upper aqueous phase was transferred to a fresh tube and an equal volume of phenol:chloroform:isoamylalcohol (25:24:1) was added. Samples were vortexed and centrifuged as previously. Total RNA in the upper aqueous phase was precipitated by addition of 100 mM sodium acetate and three volumes of ethanol. Total RNA was extracted from human cells using TRI reagent (Sigma) according to the manufacturer’s instructions.

Total RNA was separated on 1.2% denaturing (glyoxal) agarose gels then transferred to nylon membranes by vacuum blotting. RNA was UV crosslinked to the membranes, which were subjected to methylene blue staining (0.03% methylene blue (w/v) in 0.3 M sodium acetate). The membranes were pre-hybridized in SES1 (0.5 M sodium phosphate pH 7.2, 7% SDS, 1 mM EDTA pH 8.0) at 37°C for 1 h before incubation with 5’ [^32^P]-labelled antisense DNA oligonucleotides (yeast SSU—5’-CGGTTTTAATTGTCCTA-3’, yeast LSU—5’- TGAGAAGGAAATGACGCT-3’, yeast scR1–5’- ATCCCGGCCGCCTCCATCAC-3’, yeast 18S 5’-GAACCAAACGTCCTATTCTATTATTC-3’, yeast 25S 5’-CTCCGCTTATTGATATGC-3’, human SSU– 5’- CCTCGCCCTCCGGGCTCCGTTAATGATC-3’, human LSU– 5’- GAGTCCGCGGTGGAG-3’, human actin 5’- AGGGATAGCACAGCCTGGATAGCAAC-3’, human 18S 5’-TGTTACGACTTTTACTTCCTCTAGATAGTC-3’, human 28S 5’-CCCGTTCCCTTGGCTGTGGTTTCGCTAGATA-3’) diluted in SES1 overnight at 37°C. Excess probes were removed by washing and membranes were exposed to phosphorimager screens. Radioactive signals were detected using a Typhoon FLA9500.

### Western blotting

Yeast cells harboring different αSyn constructs were grown in selective SC medium containing 2% raffinose overnight and transferred to 2% galactose-containing medium at *A*_*600*_ = 0,1 for induction of αSyn expression for 6 h. Cells were lysed with glass beads (∅ 0.25–0.5 mm, Carl Roth GmbH, Germany) in a buffer containing 1 mM EDTA pH 7.5, 5 mM DTT, 50 mM Tris-HCl pH 7.5, 20 μl/ml protease inhibitor cocktail (Complete, EDTA-free, Roche Diagnostics GmbH, Germany) at 4°C, and centrifuged at 13000 rpm for 15 min to remove glass beads and large cell debris. Protein concentration was determined using Bradford protein assay and the protein samples were denatured in SDS-sample buffer (50 mM Tris-HCl pH 6.8, 3% (v/v) β-mercaptoethanol, 2% (w/v) SDS, 1% (v/v) glycerol, 0.006% (w/v) bromophenol blue). For electrophoretic separations of the protein, equal amounts of protein extracts were subjected to 12% SDS-polyacrylamide-gel and transferred onto a nitrocellulose membrane. Blots were blocked in 5% skin milk powder in TBST buffer for 1 h and incubated with primary antibody diluted in TBST buffer with 5% milk powder overnight. αSyn rabbit antibody (Santa Cruz Biotechnology) was used for detection of αSyn in yeast. After three 10 min washes with TBST buffer, blots were incubated with secondary antibodies such as anti-mouse or anti-rabbit IgG conjugated to peroxidase for 2 h at RT. After three 10 min washes with TBST, chemiluminescent reaction was performed with a detection substrate (44 μl 90 mM paracoumaric acid, 100 μl 2.5 M luminol, 6.2 μl H_2_O_2_, 2 ml 1 M Tris pH 8.5, 18 ml H_2_O). Pixel density values for Western blot quantifications were obtained from TIFF files generated from digitized x-ray films (Kodak, USA) and analyzed with the ImageJ software (NIH, Bethesda, USA). Sample density values were normalized to the corresponding loading control. For quantification of the signals, at least three independent experiments were performed.

Dot-blot analysis was performed with equal volumes of protein samples from end-point aggregation reactions. 10 μl and 2 μl from each sample were applied on a nitrocellulose membrane, samples were vacuum-filtered and allowed to dry. The membranes were blocked in 5% skin milk powder in TBST buffer for 1 h, incubated with rabbit A11 anti-oligomer antibody for 1 h and processed as above. The amount of protein adsorbed onto the membrane was assessed using anti-αSyn antibody that recognizes all protein species.

### Sucrose density gradient centrifugation

Sucrose density gradient centrifugation was performed as described previously [60]. In brief, αSyn expression was induced for 6 h in SC selection medium supplemented with 2% galactose. Whole cell extracts were prepared from 500 ml cells in mid-log phase. Cells were pelleted and resuspended in 1.5 volumes of extraction buffer (50 mM Tris-HCl pH 7.5, 100 mM NaCl, 5 mM MgCl_2_, 1 mM DTT, 5 μl RNasin, 20 μl/ml protease inhibitor cocktail). The cell suspension was frozen by dropping into liquid nitrogen and the frozen cells were broken by grinding in a pre-cooled mortar in liquid nitrogen. The cell lysate was cleared by centrifugation at 20000 x g for 15 min at 4°C. 200 μl of clarified lysate was loaded on top of a 12 ml 10–45% sucrose gradient, prepared by overlaying 10% and 45% sucrose solutions in 50 mM Tris-HCl pH 7.5, 100 mM NaCl, 5 mM MgCl_2_, 1 mM DTT and linear gradient formation using a GradientMaster (Biocomp). Centrifugation was performed for 16 h at 23500 rpm in a SW40Ti rotor at 4°C. Fractions of 530 μl were collected from the top of the gradient. The proteins from the gradient fractions were precipitated with 125 μl of 100% TCA. The samples were vortexed and placed on ice for 20 min, followed by centrifugation for 20 min at 20000 x g at 4°C. Protein pellets were washed with 1 ml cold acetone, dried for 5 min at 37°C and resuspended in 30 μl SDS-sample buffer for analysis by western blotting.

### Preparation of nuclei from yeast

Yeast nuclei were isolated with Yeast Nuclei Isolation Kit (BioVision, USA). Cells, corresponding to OD = 50 were harvested by centrifugation and used for the nuclei preparation according to the manufacturer’s instructions. The quality of the nuclei was examined by fluorescence microscopy using 4 μl nuclei stained with 4 μl of DAPI (1 μg/ml) and the nuclei were stored at -80°C. For Western blot analysis, 20 μl nuclei were resuspended in HU buffer (200 mM Tris buffer, pH 6.8, 8 M urea, 5% w/v SDS, 1 mM EDTA, 100 mM DTT) and heat-denatured at 65°C for 10 min.

### Protein purification of αSyn

The expression and purification of αSyn was performed as previously described [61]. Briefly, *E*. *coli* BL21 (DE3) cells were transformed with pET22b-αSyn construct, and expression of 500 ml LB culture was induced with isopropyl β-D-1-thiogalactopyranoside (IPTG) with final concentration of 1 mM at OD = 0.3 at 37°C overnight. Cells were pelleted, frozen at -80°C, and re-suspended in lysis buffer (750 mM NaCl, 10 mM Tris pH 8.0, 1 mM EDTA, 20 μl/ml protease inhibitor cocktail). Cells were lysed by sonication on ice (5 times, 30 s each step, cool down for 1 min after each sonication step), heated at 95°C for 15 min and then centrifuged at 13000 rpm at 4°C for 20 min. The supernatant was dialyzed overnight at 4°C against dialysis buffer (50 mM NaCl, 10 mM Tris pH 7.6, 1 mM EDTA). αSyn was purified on two HiTrap Q FF 1 ml anion exchange columns (GE Healthcare, USA) in 25 mM Tris pH 7.7 with a NaCl gradient from 0 to 600 mM. αSyn fractions were collected as judged by 12% SDS-polyacrylamide gel electrophoresis. αSyn was further purified by size-exclusion chromatography on a Superdex 75 26/600 prep grade 120 ml column (GE Healthcare, USA) in SEC buffer (100 mM NaCl, 25 mM HEPES, 1 mM DTT, pH 8.0). The purification of αSyn was confirmed by SDS-PAGE and Western blotting analysis, and proteins were stored at -80°C.

### Protein purification of Dbp4 and DDX10

*E*. *coli* BL21 (DE3) cells were transformed with pET22b-*DBP4*-His_6_ or pET22b-*DDX10*-His_6_ constructs and expression of 2 l LB culture was induced with IPTG with final concentration of 1 mM at OD = 0.3. *DBP4* expression was induced at 20°C overnight and *DDX10* at 37°C for 3 h. The cells were collected and re-suspended in 80 ml lysis buffer (500 mM NaCl, 2 mM MgSO_4_, 50 mM HEPES pH 8.0, 1 mM protease inhibitor mix). The cells were lysed by sonication on ice (5 times, 30 s each time, cool down for 1 min after each sonication step) and then centrifuged at 13000 rpm at 4°C for 30 min. The supernatant was mixed with Ni^2+^-NTA beads (Qiagen, Germany) and rotated for 1.5 h at 4°C. The beads were collected and washed two times with washing buffer (lysis buffer supplemented with 20 mM imidazole). Then the proteins were eluted with elution buffer (lysis buffer supplemented with 250 mM imidazole). The proteins were further purified by size-exclusion chromatography on a Superdex 75 26/600 prep grade 120 ml column (GE Healthcare) in SEC buffer (250 mM NaCl, 20 mM HEPES, 1 mM DTT, pH 8.0). The purification of the proteins was confirmed by SDS-PAGE and Western blotting analysis.

### Pull-down assay

Purified αSyn protein was mixed with equal amount of Dbp4 or DDX10 His6-tagged proteins in SEC buffer (250 mM NaCl, 20 mM HEPES, pH 8.0) and the samples were incubated for 30 min at 4°C. As control, the proteins were incubated alone. The samples were mixed with Ni^2+^-NTA beads (Qiagen, Germany) and rotated for 15 min at 4°C. The beads were collected and washed three times with washing buffer (SEC buffer supplemented with 20 mM imidazole). Then the proteins were eluted with elution buffer (SEC buffer supplemented with 250 mM imidazole). Western blotting was performed with αSyn mouse antibody (BD Transduction Laboratory) or His6-antibody (ThermoFisher) with 10% of the input fractions, 10% of the third washing fractions and 10% of the elution fractions.

### Fluorescence microscopy and quantifications

Yeast cells harboring αSyn-expressing plasmids were pre-grown in selective SC medium containing 2% raffinose at 30°C overnight and transferred into galactose-containing SC medium at *A*_*600*_ = 0.1 for induction of αSyn expression for 6 h. Fluorescence images were obtained with 63x or 100x magnification using a Zeiss Observer. Z1 microscope (Zeiss) equipped with a CSU-X1 A1 confocal scanner unit (YOKOGAWA), QuantEM:512SC digital camera (Photometrics) and SlideBook 6.0 software package (Intelligent Imaging Innovations). Nuclei were stained with DAPI (4,6-Diamidin-2-phenylindol) at final concentration of 2.5 μg/ml for 30 min before harvesting and microscope observation. For quantification of inclusion formation at least 200 cells were counted per strain and experiment. The number of cells displaying αSyn inclusions was referred to the total number of counted cells. Quantification of the GFP intensity of the nucleolus and nucleoplasm was performed using SlideBook 6.0 segmentation software tool of DAPI stained yeast cells. The nucleolar region was determined by thresholding based on GFP image intensities. The nucleoplasm region was defined from DAPI staining using the freehand tool and excluded the nucleolar region. The fluorescence intensity of the BiFC signal/cell was quantified by measuring the intensity per cell. All fluorescence measurements were performed after subtraction of the background fluorescence.

HEK cells were visualized using the Olympus IX81-ZDC microscope system with a 20×objective. Random fields were taken out of two independent experiments. The mean intensity was measured using the Olympus ScanR Image Analysis Software.

### Promoter shut-off assay

Yeast cells harboring αSyn-expressing plasmids were pre-grown overnight in selective SC medium containing 2% raffinose and shifted to 2% galactose-containing selective SC medium at *A*_*600*_ = 0.1 for induction of αSyn expression for 4 h. Afterwards, cells were pelleted, washed two times with water and shifted to SC medium supplemented with 2% glucose to shut-off the *GAL1* promoter. The cells were visualized by fluorescence microscopy after 2 h, 4 h and 6 h. The reduction of number of cells displaying αSyn inclusions was recorded and plotted on a graph.

### Thioflavin T assay

Thioflavin T (ThT) assay was performed as described [62] with modifications. αSyn was incubated at a final concentration of 15 μM in a buffer containing 150 mM NaCl, 1 x PBS, 1 mM EDTA, 0.002% SDS and 10 μM Thioflavin T, either alone or in presence of 2.5 μM Dbp4, DDX10 or BSA in a final volume of 100 μl per well. The samples were incubated at 37°C for 80 h with continuous shaking in a black 96-well plate, covered with adhesive plate sealer. Fibril formation was monitored by fluorescence every 2 min, with excitation at 440 nm and emission at 480 nm in a fluorescence plate reader (Tecan Infinite M200, Switzerland). All experiments were carried out in triplicates.

### Transmission electron microscopy

αSyn aggregation products were analyzed by negative staining and transmission electron microscopy. Carbon-coated mica (3x3 mm) was introduced onto a sample drop for 1 min in order to absorb the proteins on the surface. For washing, the mica surface was placed on a water drop for 3 s to remove salts and buffer. Staining of the sample was performed by slowly immersing the coated mica into phosphotungstic acid (pH 7.0, 3%), and floating the carbon sheet off the mica onto the surface. Electron microscope grids were placed on the surface for 5 s, resulting in the entire grid area being covered with the proteins attached by carbon. The grid was removed from the droplet, and excess fluid was blotted by touching the grid vertically with a piece of filter paper. After drying, the negative stained samples were imaged using Jeol EM 1011 transmission electron microscope (Jeol, Eching, Germany).

### Immunoelectron microscopy

Immunogold labeling of yeast cells was performed as described with modifications [63]. Yeast cells were pre-grown in selective SC medium containing 2% raffinose at 30°C overnight and transferred into galactose-containing SC medium at *A*_*600*_ = 0.1 for induction of αSyn and *DBP4* expression for 6 h. Cells, equivalent to OD_600_ = 1 were harvested and resuspended in an equal volume of double-strength fixative containing 4% paraformaldehyde (PFA), 0.4% glutaraldehyde (GA) in 0.1 M PHEM buffer (120 mM PIPES, 50 mM HEPES, 4 mM MgCl_2_, 20 mM EGTA, pH 6.9). The cell suspension was incubated for 10–20 min at RT on a roller. The fixative was replaced with fresh standard strength fixative (2% PFA, 0.2% GA in 0.1 M PHEM buffer) and fixation was continued for 3 h. The cell pellet was washed 3 times with 0.1 M PHEM buffer, resuspended in 1% periodic acid in 0.1 M PHEM buffer and incubated on a roller for 1 h. Cells were resuspended in warm 10% gelatin (in PHEM buffer), incubated for 10 min at 37°C. The cells were pelleted, and the gelatin was let to solidify on ice. Small blocks of gelatin embedded cells were cut (1 mm^3^) and immersed in 2.3 M sucrose in 0.1 M PHEM buffer overnight. The blocks were mounted on aluminum pins and frozen in liquid N_2_. Sections were prepared with a cryo-ultramicrotome (UC6, Leica Microsystems, Vienna, Austria) and a 35° cryo-immuno diamond knife (Diatome, Biel, Switzerland) at -110°C and picked-up with a 1:1 mixture of 2.3 M sucrose and 2% methylcellulose. Before immunolabeling gelatin was removed by placing grids on warm PBS for 20 min. Immunolabelling was performed as described (Griffith et al, 2008) by using rabbit αSyn-antibody (AnaSpec) (1:10) and rat GFP-antibody (1:50) and protein-A coupled to colloidal gold of different size. Gold particles: GFP = 15 nm; αSyn = 10 nm. Control immunolabeling was performed under the same conditions by omitting the primary antibodies. The gold particles in the nucleolus and in the nucleoplasm were counted manually. Gold particles at the border and nearby to the dense nucleolar region were assigned to nucleolus, and gold particles far away from the dense region were assigned to nucleus. The areas of the nucleolus and nucleus were determined using ImageJ. The background labeling density was estimated by the count of gold particles/μm^2^ in the cytoplasm and subtracted from the nuclear counts/μm^2^. For evaluation of Dbp4 and αSyn colocalization, co-immunolabeled cells were used. The 15 nm gold particles (GFP-labeling) that are in proximity to 10 nm gold particles (αSyn-labeling) were counted in the nuclear and cytoplasmic region and referred to the total number of 15 nm gold particles (GFP-labeling) per cell. Regions in the plasma membrane with dark staining, where the gold particles cannot be unambiguously attributed to one of the two types, were excluded from the evaluation.

### Cell culture

Human Embryonic Kidney 293 (HEK) cells were maintained in Dulbecco’s Modified Eagle Medium (DMEM, Life Technologies- Invitrogen, USA) supplemented with 10% Fetal Bovine Serum Gold (FBS) (PAA, Germany) and 1% Penicillin-Streptomycin (PAN, Germany). The cells were grown at 37°C in an atmosphere of 5% CO2.

### Cell transfection

The cells were plated in 12-well plates (Costar, Corning, New York, USA) the day before. Equimolar amounts of the plasmids and Metafectene were used (Biotex, Germany) following the manufacturer’s instructions. Twenty-four hours after transfection the cells were fixed for further analysis.

### Immunocytochemistry

Twenty-four hours after transfection, cells were fixed with 4% paraformal- dehyde (PFA) for 10 min at RT, followed by a permeabilization step with 0.1% Triton X-100 (Sigma-Aldrich, USA) for 20 minutes at RT. The cells were blocked in 1.5% normal goat serum (PAA, Germany) / PBS for 1 hour, followed by incubation with primary antibody (Syn1, 1:1000, BD Transduction Laboratory, USA) overnight. After washing the cells with 1xPBS, the cells were incubated with secondary antibody (Alexa Fluor 488 donkey anti-mouse IgG; Life Technologies-Invitrogen, USA) for 2 hours at RT. Finally, cells were stained with Hoechst 33258 (Life Technologies-Invitrogen, USA) (1:5000 in 1xPBS) for 5 minutes and maintained in PBS for epifluorescence microscopy.

### Quantification and statistical analysis

Data were analyzed using GraphPad Prism 5 software (San Diego, California, USA) and were presented as mean ± SEM of at least three independent experiments. P value < 0.05 was considered to indicate a significant difference.

## Supporting information

S1 Fig Random spore analysis of the genetic interactions from yTHC-screen. Examples of random spore analysis performed with spores, saved from the sporulation step of SGA procedure. **(A)** Spores were suspended in sterile water and plated on the following media: 1) SD-Ura/His/Arg/Lys + canavanine + thyalysine + glucose (selection for all haploid spore products of *Mata*); 2) SD- Ura/Leu/His/Arg/Lys + canavanine + thyalysine + G418 + glucose (selection for haploid TetO_7_-allele and αSyn-allele); 3) SD-Ura/Leu/His/Arg/Lys + canavanine + thyalysine + G418 + doxycycline + glucose (selection for haploid TetO_7_-allele and αSyn-allele, shut-off of Tet-promoter); 4) SD-Ura/Leu/His/Arg/Lys + canavanine + thyalysine + G418 + galactose (selection for haploid TetO_7_-allele and αSyn-allele, shut-on of GAL1-promoter); 5) SD-Ura/Leu/His/Arg/Lys + canavanine + thyalysine + G418 + doxycycline + galactose (selection for haploid TetO_7_-allele and αSyn-allele, shut-on of GAL1-promoter; shut-off of Tet-promoter). The plates were incubated at 30°C for 3 days and scored by comparison of the cell growth on the five plates. **(B-H)** Examples of different types of interactions.
(TIF)

S2 Fig Growth effect on yeast cells due to interaction between αSyn and *Tet*-alleles of essential genes. Yeast cells expressing *GAL1*-driven αSyn and the corresponding *Tet*-alleles of the indicated essential genes were spotted in 10-fold dilutions on selection plates containing glucose (αSyn-OFF) or galactose (αSyn-ON) in presence (*Tet*-OFF) or absence (*Tet*-ON) of 10 μg/ml doxycycline that represses *Tet*-promoter. Empty vector was used as a control. The plates were incubated at 30°C for 4 days. In presence of αSyn, growth enhancement upon downregulation of *Tet*-ORFs indicates a putative target gene (*SCP110*, *DBP4*). *RNA1* and *RPA43* reveal better growth upon downregulation of the essential gene upon αSyn expression in comparison with absence of αSyn expression. Synthetic sick phenotype upon downregulation of *Tet*-ORFs indicates protective genes (the rest).
(TIF)

S3 Fig Downregulation of the essential genes changes the inclusion formation of αSyn. **(A)** Fluorescence microscopy of the indicated strains with *Tet*-alleles of essential genes after 6 h induction of αSyn expression. *Tet*-promoter was downregulated by addition of doxycycline to the growth medium simultaneously with the induction of αSyn expression. Control: αSyn expression in the background strain of yTHC collection. (**B)** Quantification of the number of cells with inclusions. “0” indicates cells without inclusion. Significance of differences was calculated with t-test (*p < 0.05; **p < 0.01; ***p < 0.001; ****p < 0.0001; n = 3).
(TIF)

S4 Fig Different impacts of overexpression of *RCL1*, *NOP4* or *DBP4* on αSyn-induced toxicity. **(A)** Growth assay of yeast cells expressing *GAL1*-driven αSyn-GFP from three genomic copies, either alone (αSyn) or in presence of *RCL1* overexpression (oe) from 2μ plasmid, driven by *GAL1* promoter (αSyn + Rcl1oe). The isogenic background strain W303 was transformed with empty vector (Control) or with 2μ plasmid overexpressing *RCL1* (Rcl1oe). Cells were spotted in tenfold dilutions on selection plates containing non-inducing glucose (*GAL1*—OFF) or galactose (*GAL1*—ON). **(B)** Growth assay of yeast cells expressing *GAL1*-driven αSyn-GFP from three genomic copies, either alone (αSyn) or in presence of *NOP4* overexpression (oe) from 2μ plasmid, driven by *GAL1* promoter (αSyn + Nop4oe). The isogenic background strain W303 was transformed with empty vector (Control) or with 2μ plasmid overexpressing *NOP4* (Nop4oe). Cells were spotted in tenfold dilutions on selection plates containing non-inducing glucose (*GAL1*—OFF) or galactose (*GAL1*—ON). **(C)** Relative mRNA expression level of *DBP4* in BY4741 and DAmP*-DBP4* strain determined by qRT-PCR in presence or absence of αSyn expression. Expression of αSyn upregulates the mRNA level of *DBP4* in BY4741 strain. mRNA level of *DBP4* in DAmP strain is significantly decreased compared to the wild type BY4741 background strain. Significance of differences was calculated with t-test (*p < 0.05; **p < 0.01; ****p < 0.0001, n = 4). **(D)** Growth assay of yeast cells expressing *GAL1*-driven αSyn-GFP from 2μ plasmid or empty vector (EV) as a control in DAmP-*Dbp4* strain or the isogenic background BY4741. Cells were spotted in tenfold dilutions on selection plates containing non-inducing glucose (*GAL1*—OFF) or galactose (*GAL1*—ON).
(TIF)

S5 Fig Overexpression of Dbp4 enhances the toxicity of A30P in yeast. **(A)** *DBP4*-expressing vector or empty vector were transformed in yeast strains, harboring three copies of *GAL1*-A30P-GFP, stably integrated in the genome, or overexpressed from 2μ plasmid (oe) in W303 strain. W303 was transformed with empty vector as a control. Yeast cells were spotted in tenfold dilutions on selection plates, supplemented with glucose (*GAL1*—OFF) or galactose (*GAL1*—ON). **(B)** Immunoblotting analysis of A30P-GFP protein levels of cells, expressing A30P-GFP from 2μ plasmid in presence or absence of *DBP4* overexpression. GAPDH antibody was used as a loading control. **(C)** Densitometric analysis of the immunodetection of A30P-GFP relative to GAPDH loading control. **(D)** Fluorescence microscopy of yeast cells, expressing A30P-GFP from 2μ plasmid in presence or absence of *DBP4* overexpression. Cells were imaged 6 h after induction of protein expression in galactose-containing medium. Scale bar = 5 μm. **(E)** Quantification of the percentage of cells displaying A30P-GFP inclusions. Significance of differences was calculated with t-test (n.s., n = 3).
(TIF)

S6 Fig The toxicity enhancement effect of Dbp4 overexpression is independent of its ATPase or helicase activity. **(A)** Domain structure of Dbp4. Indicated are the DEAD-box domain, Helicase superfamily C-terminal domain (Helicase_C domain) and domain of unknown function (DUF). The arrowheads indicate the positions of the amino acid exchanges. **(B)** Spotting assay of W303 cells, expressing *GAL1*-driven αSyn, Dbp4 or Dbp4-mutants from 2μ plasmid. Dbp4 (GRT) indicates K91R substitution in Walker A Motif I (GKT). Dbp4 (AAA) indicates S225A/T227A double substitution in Motif III (SAT). Cells were spotted in tenfold dilutions on selection plates containing non-inducing glucose (*GAL1*—OFF) or galactose (*GAL1*—ON). Cells transformed with empty vector were used as a control. **(C)** Densitometric analysis of the detected levels of mature 25S and 18S rRNA from Fig 3F. Left panel—ratio of 18S to 25S in presence and absence of αSyn or Dbp4oe. Right panel: 25S or 18S levels relative to scR1 loading control. Cells transformed with empty vector were used as a control. **(D)** Densitometric analysis of the detected levels of mature 25S and 18S rRNA. Ratio of 18S to 25S of cells expressing (+) or not (-) αSyn in the *Tet*-*DBP4* strain in the presence (*Tet*-OFF) or absence (*Tet*-ON) of doxycycline (right panel). Significance of differences was calculated with t-test (*p < 0.05). **(E)** Ratio of the detected levels of 28S and 18S rRNA species in HEK293 cells stably transfected with EGFP (control) or αSyn -EGFP.
(TIF)

S7 Fig BiFC competition assay. **(A)** Fluorescence microscopy of yeast cells, expressing the indicated constructs 6 h post induction. Scale bar = 5 μm. **(B)** BiFC competition assay evaluation. Fluorescent cells from (A) were counted and referred to the total number of cells. **(C)** Western blot analysis of cells from (A). αSyn antibody detects VN-αSyn, αSyn-VC and αSyn (no tag). GFP antibody detects VC but not VN. GAPDH antibody is used as a loading control. **(D)** Comparison of the BIFC signal intensities of cells expressing VN-αSyn + αSyn-VC or VN-αSyn + Dbp4-VC (n = 30).
(TIF)

S8 Fig Impact of αSyn-mCherry expression on the cellular localization of Dbp4, Rcl1 or Nop4. **(A)** Fluorescence microscopy of cells expressing *DBP4*-GFP from its native promoter or overexpressed from 2μ vector in presence or absence of αSyn-mCherry. Shown are cells with plasma membrane localization of αSyn-mCherry or inclusions at the plasma membrane. **(B)** Fluorescence microscopy of Rcl1-GFP or Nop4-GFP **(C)** expressing cells driven by endogenous promoters and expressing αSyn-mCherry or empty vector (EV) as a control. Expression of αSyn was induced for 6 h in galactose-containing medium. Scale bar = 1 μm.
(TIF)

S9 Fig Pull-down assay with purified αSyn and His6-tagged Dbp4 or DDX10 proteins. Dbp4 and DDX10 were expressed as 6xHis fusions in *E*. *coli* and purified. αSyn was purified without a tag. His6-tagged proteins were incubated alone or with αSyn for 30 min and subsequently bound to Ni-NTA beads. αSyn alone served as a control. After washing the samples were eluted. Western blot analysis was performed using αSyn and His6 antibodies with 10% of the input, 10% of the last washing fraction and 10% of the elution fraction 1 (E1) and elution fraction 2 (E2).
(TIF)

S1 Table Identified genetic interactions between αSyn and *Tet*-alleles of essential genes. The first column indicates the standard gene name. The second column indicates the human orthologs. Type and strength correspond to observed genetic interactions in spotting assays after downregulation of the essential genes resulting in synthetic-sick (**-**) phenotype (protective gene) or growth enhancement (**+**) phenotype (drug target). Brief description of the protein function is deduced from the *Saccharomyces* genome database (SGD). Genes were classified in one functional category according to their function or biological process using Gene Ontology (GO) annotations, generated by Saccharomyces Genome Database (SGD) Gene Ontology Slim Mapper, FunSpec webserver and manual curation.
(DOCX)

S2 Table Observed genetic interactions between αSyn and the hypomorphic alleles of essential genes from the DAmP collection. The first column indicates the standard gene name; the second column indicates the systematic gene name. The third column gives a brief description of the protein’s function according to *Saccharomyces* genome database (SGD). “Score” represents the fitness of growth, calculated as ratio of the colony area of αSyn expressing strains to corresponding vector control on plates with galactose-inducing medium. The value is mean of four independent screens. The table lists hits with score < 0.85 and standard deviation < 0.05. Genes identified in both yTHC and DAmP screen are indicated by *. 76 out of 84 genes have human orthologs, indicated in the last column.
(DOCX)

S3 Table Distribution of the identified genes from DAmP screen into functional categories. Genes were classified in one category according to their function or biological process using Gene Ontology (GO) annotations, generated by Saccharomyces Genome Database (SGD) Gene Ontology Slim Mapper, FunSpec webserver and manual curation.
(DOCX)

S4 Table Subcellular localization of the gene products of yTHC gene modifiers. The subcellular localization and enrichment analysis were carried out using FunSpec webserver.
(DOCX)

S5 Table Subcellular localization of the gene products of DAmP screen modifiers. The subcellular localization and enrichment analysis were carried out using FunSpec webserver.
(DOCX)

S6 Table Reagents and Tools. (DOCX)
