# Supplementary figures and images for: DEAD-box RNA helicase Dbp4/DDX10 is an enhancer of α-synuclein toxicity and oligomerization
Source: PLoS Genet. 2021 Mar 3;17(3):e1009407. doi: 10.1371/journal.pgen.1009407 (PMC7928443; doi:10.1371/journal.pgen.1009407)

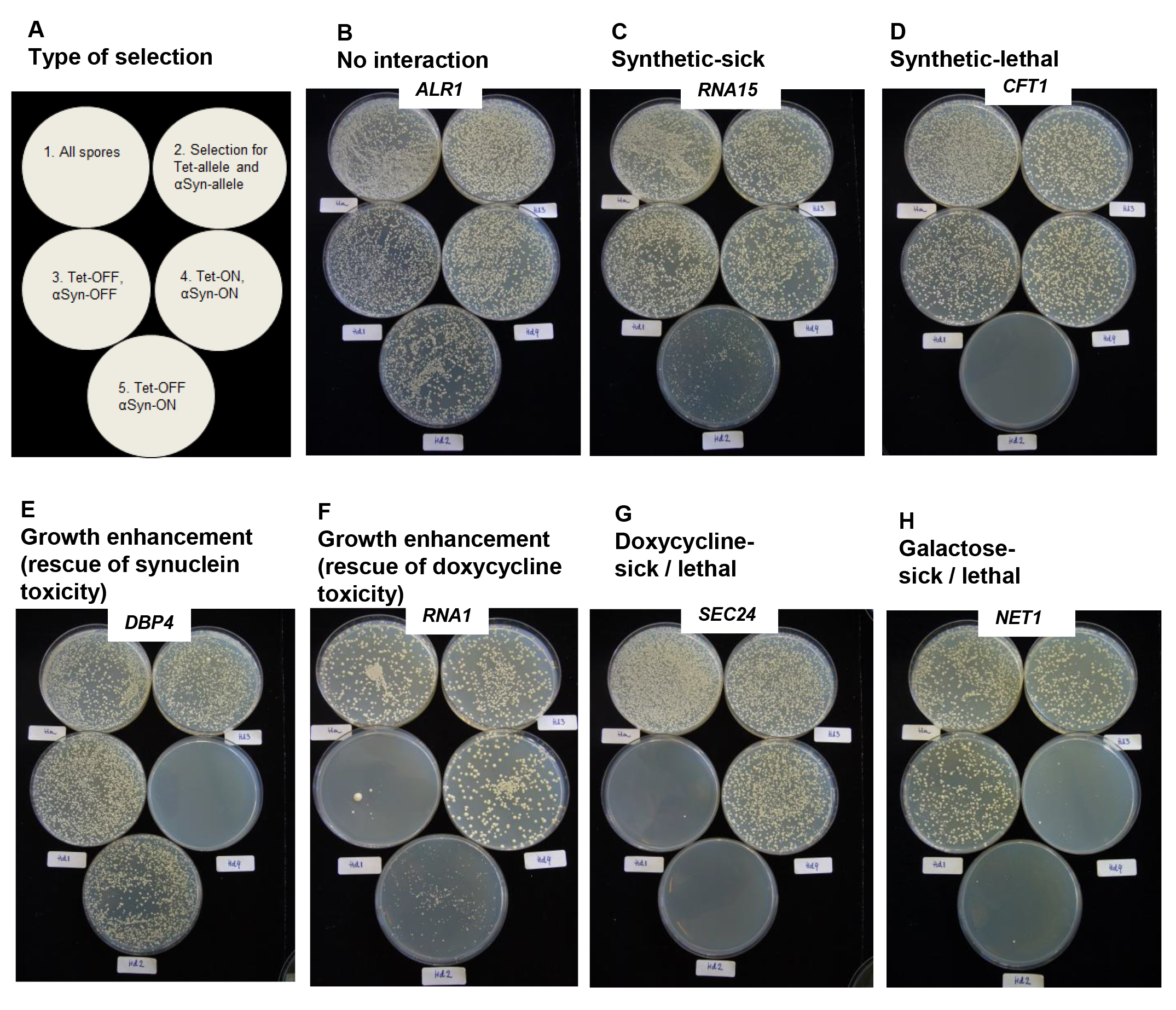

Supplement: S1 Fig — Examples of random spore analysis performed with spores, saved from the sporulation step of SGA procedure. (A) Spores were suspended in sterile water and plated on the following media: 1) SD-Ura/His/Arg/Lys + canavanine + thyalysine + glucose (selection for all haploid spore products of Mata); 2) SD- Ura/Leu/His/Arg/Lys + canavanine + thyalysine + G418 + glucose (selection for haploid TetO7-allele and αSyn-allele); 3) SD-Ura/Leu/His/Arg/Lys + canavanine + thyalysine + G418 + doxycycline + glucose (selection for haploid TetO7-allele and αSyn-allele, shut-off of Tet-promoter); 4) SD-Ura/Leu/His/Arg/Lys + canavanine + thyalysine + G418 + galactose (selection for haploid TetO7-allele and αSyn-allele, shut-on of GAL1-promoter); 5) SD-Ura/Leu/His/Arg/Lys + canavanine + thyalysine + G418 + doxycycline + galactose (selection for haploid TetO7-allele and αSyn-allele, shut-on of GAL1-promoter; shut-off of Tet-promoter). The plates were incubated at 30°C for 3 days and scored by comparison of the cell growth on the five plates. (B-H) Examples of different types of interactions. (TIF) [file pgen.1009407.s001.tif]

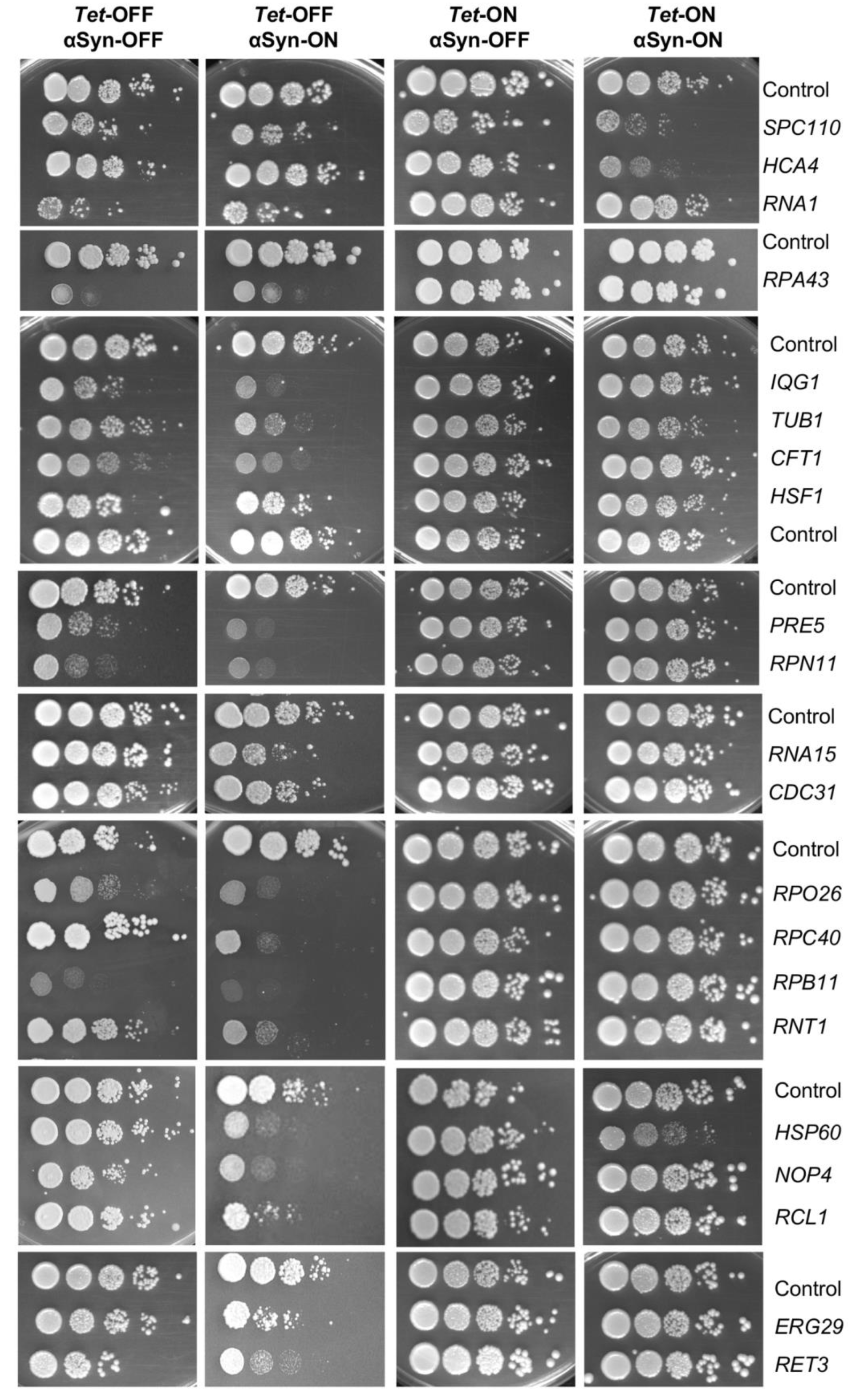

Supplement: S2 Fig — Yeast cells expressing GAL1-driven αSyn and the corresponding Tet-alleles of the indicated essential genes were spotted in 10-fold dilutions on selection plates containing glucose (αSyn-OFF) or galactose (αSyn-ON) in presence (Tet-OFF) or absence (Tet-ON) of 10 μg/ml doxycycline that represses Tet-promoter. Empty vector was used as a control. The plates were incubated at 30°C for 4 days. In presence of αSyn, growth enhancement upon downregulation of Tet-ORFs indicates a putative target gene (SCP110, DBP4). RNA1 and RPA43 reveal better growth upon downregulation of the essential gene upon αSyn expression in comparison with absence of αSyn expression. Synthetic sick phenotype upon downregulation of Tet-ORFs indicates protective genes (the rest). (TIF) [file pgen.1009407.s002.tif]

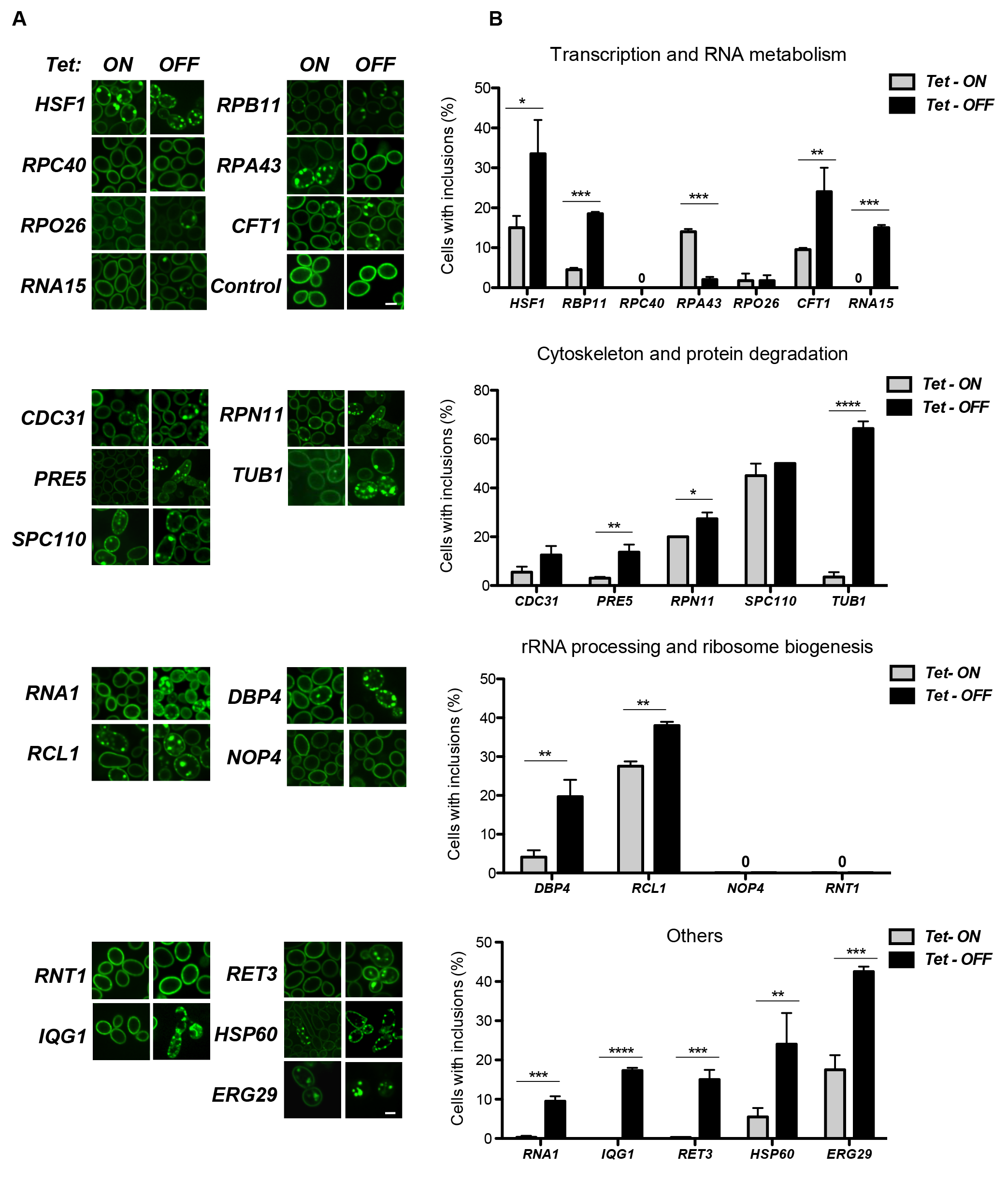

Supplement: S3 Fig — (A) Fluorescence microscopy of the indicated strains with Tet-alleles of essential genes after 6 h induction of αSyn expression. Tet-promoter was downregulated by addition of doxycycline to the growth medium simultaneously with the induction of αSyn expression. Control: αSyn expression in the background strain of yTHC collection. (B) Quantification of the number of cells with inclusions. “0” indicates cells without inclusion. Significance of differences was calculated with t-test (*p < 0.05; **p < 0.01; ***p < 0.001; ****p < 0.0001; n = 3). (TIF) [file pgen.1009407.s003.tif]

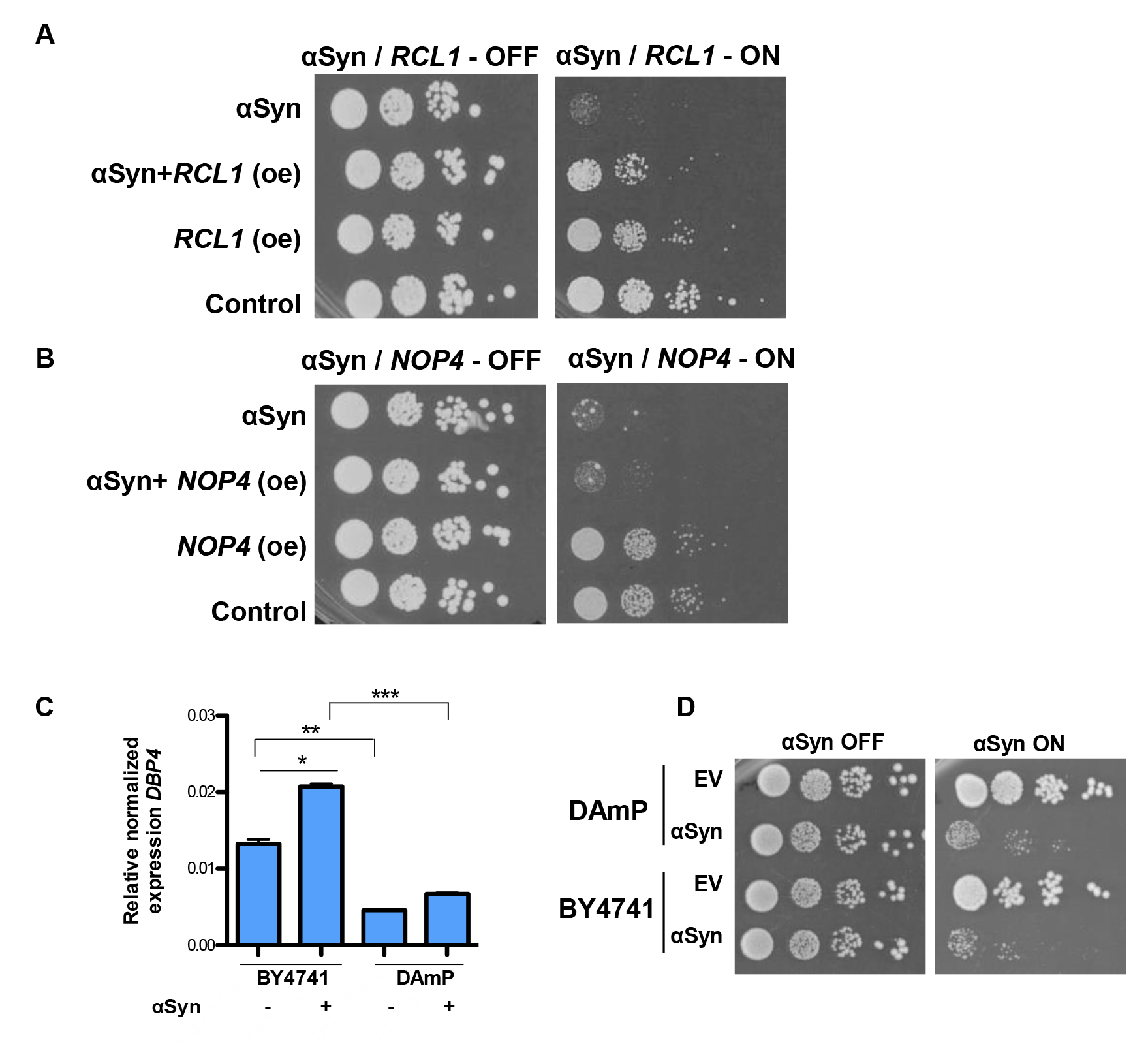

Supplement: S4 Fig — (A) Growth assay of yeast cells expressing GAL1-driven αSyn-GFP from three genomic copies, either alone (αSyn) or in presence of RCL1 overexpression (oe) from 2μ plasmid, driven by GAL1 promoter (αSyn + Rcl1oe). The isogenic background strain W303 was transformed with empty vector (Control) or with 2μ plasmid overexpressing RCL1 (Rcl1oe). Cells were spotted in tenfold dilutions on selection plates containing non-inducing glucose (GAL1—OFF) or galactose (GAL1—ON). (B) Growth assay of yeast cells expressing GAL1-driven αSyn-GFP from three genomic copies, either alone (αSyn) or in presence of NOP4 overexpression (oe) from 2μ plasmid, driven by GAL1 promoter (αSyn + Nop4oe). The isogenic background strain W303 was transformed with empty vector (Control) or with 2μ plasmid overexpressing NOP4 (Nop4oe). Cells were spotted in tenfold dilutions on selection plates containing non-inducing glucose (GAL1—OFF) or galactose (GAL1—ON). (C) Relative mRNA expression level of DBP4 in BY4741 and DAmP-DBP4 strain determined by qRT-PCR in presence or absence of αSyn expression. Expression of αSyn upregulates the mRNA level of DBP4 in BY4741 strain. mRNA level of DBP4 in DAmP strain is significantly decreased compared to the wild type BY4741 background strain. Significance of differences was calculated with t-test (*p < 0.05; **p < 0.01; ****p < 0.0001, n = 4). (D) Growth assay of yeast cells expressing GAL1-driven αSyn-GFP from 2μ plasmid or empty vector (EV) as a control in DAmP-Dbp4 strain or the isogenic background BY4741. Cells were spotted in tenfold dilutions on selection plates containing non-inducing glucose (GAL1—OFF) or galactose (GAL1—ON). (TIF) [file pgen.1009407.s004.tif]

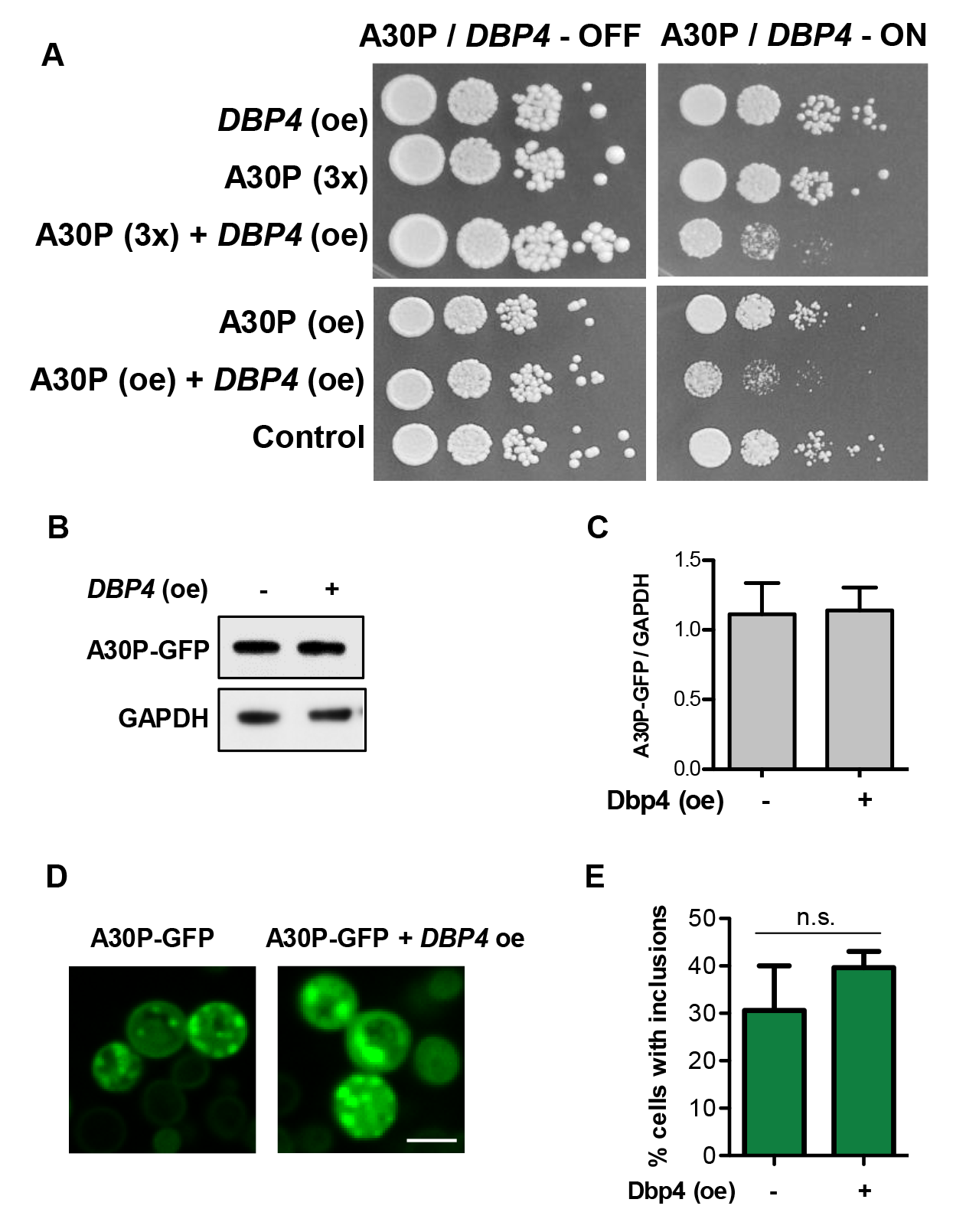

Supplement: S5 Fig — (A) DBP4-expressing vector or empty vector were transformed in yeast strains, harboring three copies of GAL1-A30P-GFP, stably integrated in the genome, or overexpressed from 2μ plasmid (oe) in W303 strain. W303 was transformed with empty vector as a control. Yeast cells were spotted in tenfold dilutions on selection plates, supplemented with glucose (GAL1—OFF) or galactose (GAL1—ON). (B) Immunoblotting analysis of A30P-GFP protein levels of cells, expressing A30P-GFP from 2μ plasmid in presence or absence of DBP4 overexpression. GAPDH antibody was used as a loading control. (C) Densitometric analysis of the immunodetection of A30P-GFP relative to GAPDH loading control. (D) Fluorescence microscopy of yeast cells, expressing A30P-GFP from 2μ plasmid in presence or absence of DBP4 overexpression. Cells were imaged 6 h after induction of protein expression in galactose-containing medium. Scale bar = 5 μm. (E) Quantification of the percentage of cells displaying A30P-GFP inclusions. Significance of differences was calculated with t-test (n.s., n = 3). (TIF) [file pgen.1009407.s005.tif]

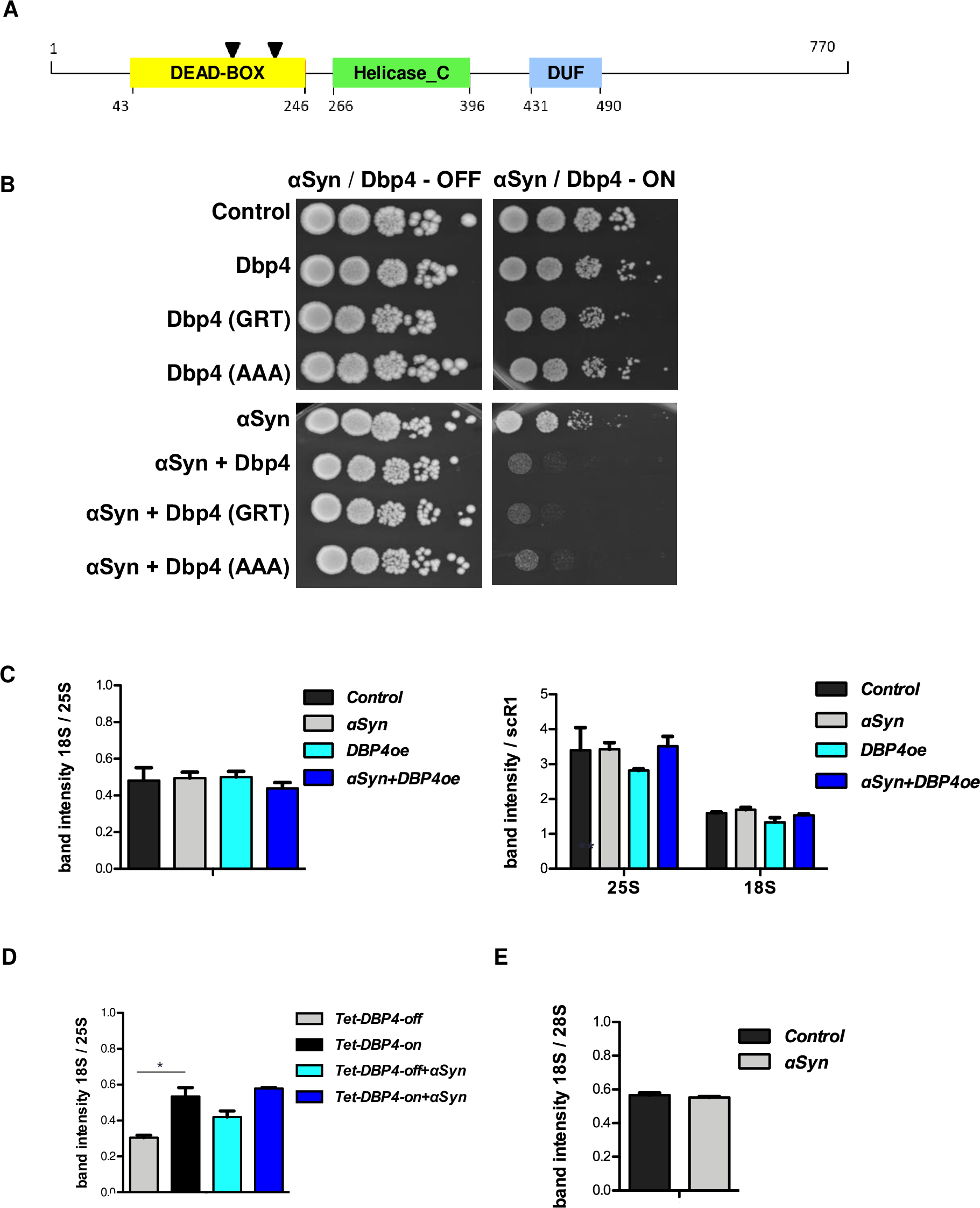

Supplement: S6 Fig — (A) Domain structure of Dbp4. Indicated are the DEAD-box domain, Helicase superfamily C-terminal domain (Helicase_C domain) and domain of unknown function (DUF). The arrowheads indicate the positions of the amino acid exchanges. (B) Spotting assay of W303 cells, expressing GAL1-driven αSyn, Dbp4 or Dbp4-mutants from 2μ plasmid. Dbp4 (GRT) indicates K91R substitution in Walker A Motif I (GKT). Dbp4 (AAA) indicates S225A/T227A double substitution in Motif III (SAT). Cells were spotted in tenfold dilutions on selection plates containing non-inducing glucose (GAL1—OFF) or galactose (GAL1—ON). Cells transformed with empty vector were used as a control. (C) Densitometric analysis of the detected levels of mature 25S and 18S rRNA from Fig 3F. Left panel—ratio of 18S to 25S in presence and absence of αSyn or Dbp4oe. Right panel: 25S or 18S levels relative to scR1 loading control. Cells transformed with empty vector were used as a control. (D) Densitometric analysis of the detected levels of mature 25S and 18S rRNA. Ratio of 18S to 25S of cells expressing (+) or not (-) αSyn in the Tet-DBP4 strain in the presence (Tet-OFF) or absence (Tet-ON) of doxycycline (right panel). Significance of differences was calculated with t-test (*p < 0.05). (E) Ratio of the detected levels of 28S and 18S rRNA species in HEK293 cells stably transfected with EGFP (control) or αSyn -EGFP. (TIF) [file pgen.1009407.s006.tif]

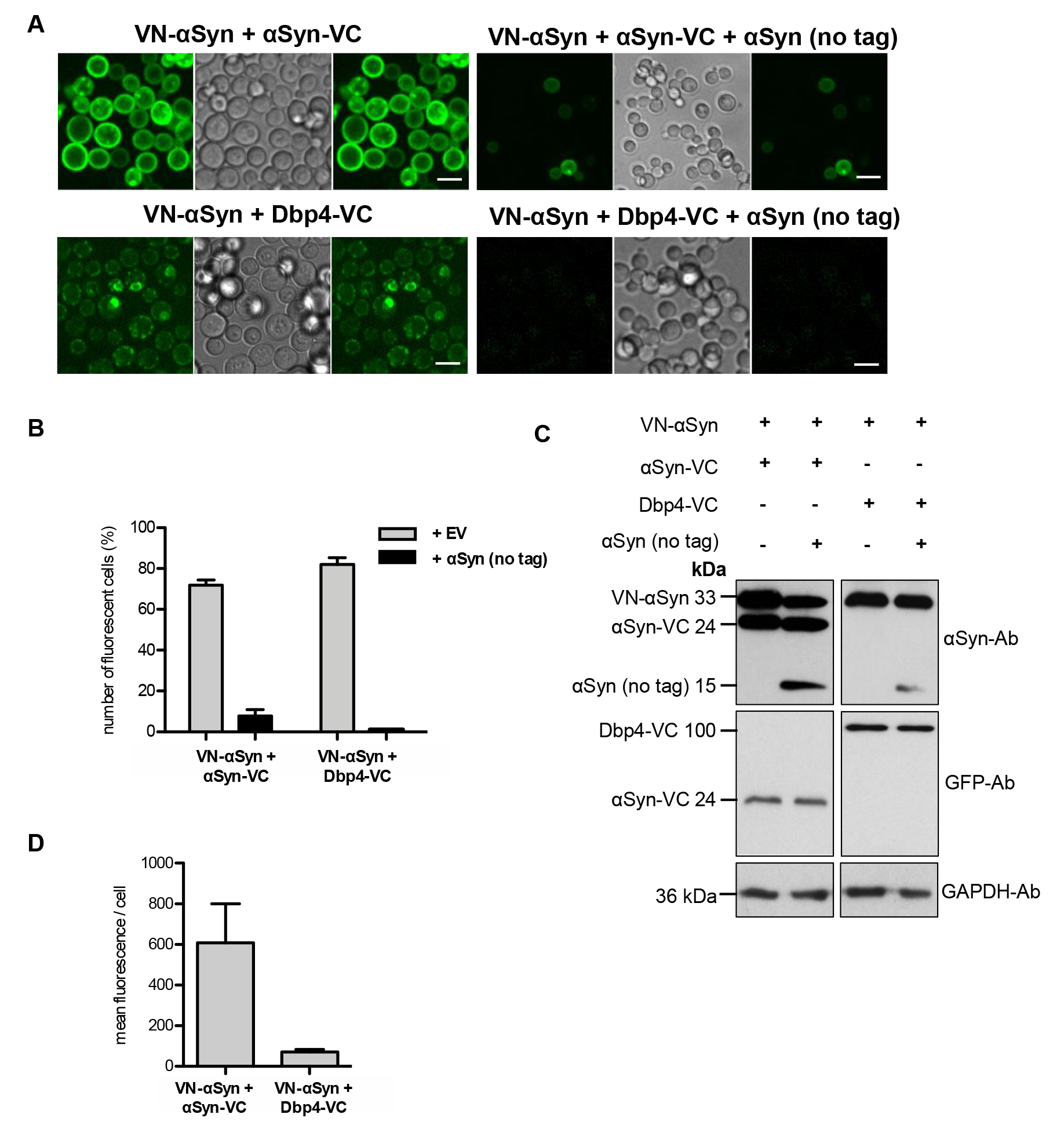

Supplement: S7 Fig — (A) Fluorescence microscopy of yeast cells, expressing the indicated constructs 6 h post induction. Scale bar = 5 μm. (B) BiFC competition assay evaluation. Fluorescent cells from (A) were counted and referred to the total number of cells. (C) Western blot analysis of cells from (A). αSyn antibody detects VN-αSyn, αSyn-VC and αSyn (no tag). GFP antibody detects VC but not VN. GAPDH antibody is used as a loading control. (D) Comparison of the BIFC signal intensities of cells expressing VN-αSyn + αSyn-VC or VN-αSyn + Dbp4-VC (n = 30). (TIF) [file pgen.1009407.s007.tif]

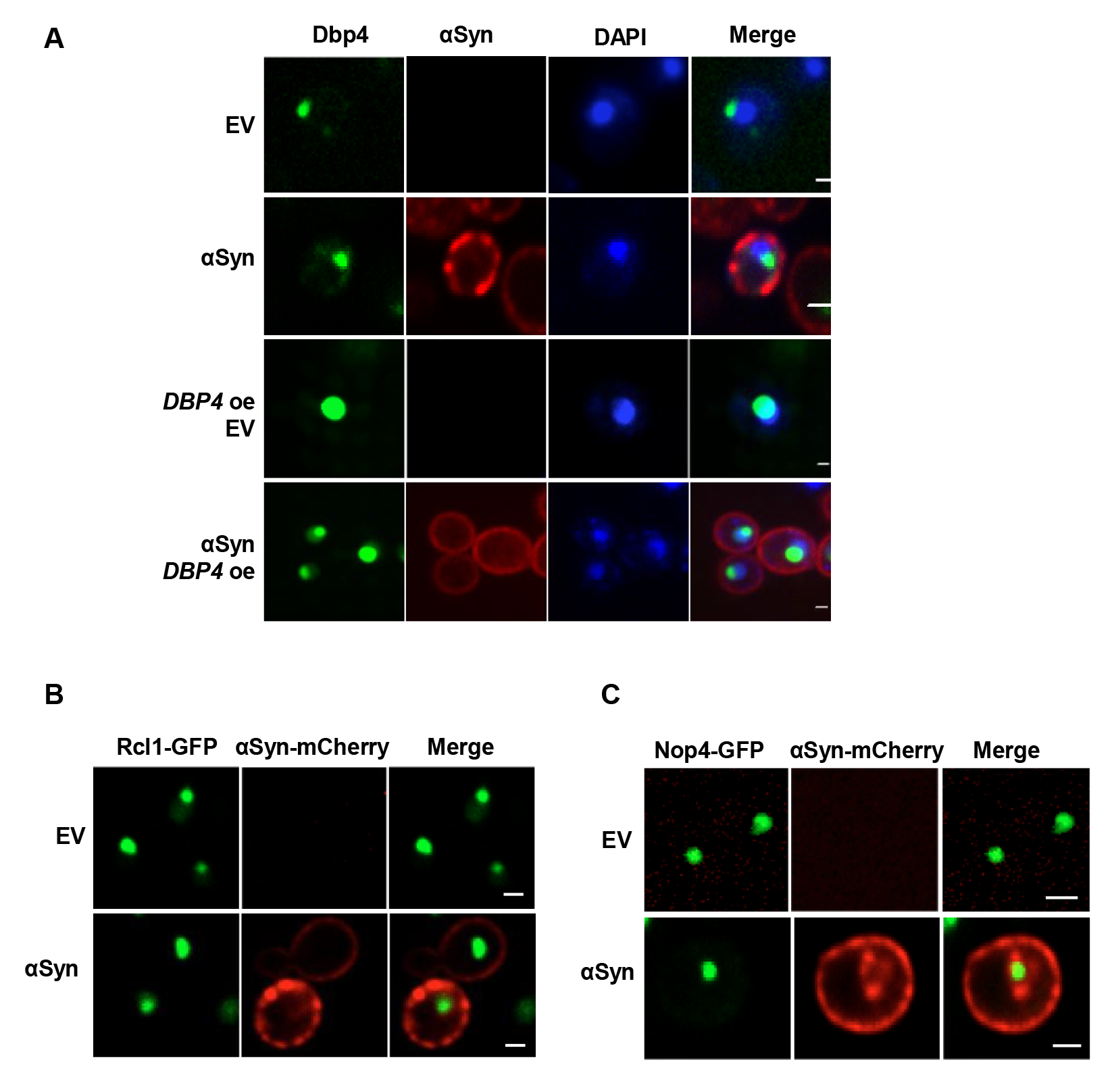

Supplement: S8 Fig — (A) Fluorescence microscopy of cells expressing DBP4-GFP from its native promoter or overexpressed from 2μ vector in presence or absence of αSyn-mCherry. Shown are cells with plasma membrane localization of αSyn-mCherry or inclusions at the plasma membrane. (B) Fluorescence microscopy of Rcl1-GFP or Nop4-GFP (C) expressing cells driven by endogenous promoters and expressing αSyn-mCherry or empty vector (EV) as a control. Expression of αSyn was induced for 6 h in galactose-containing medium. Scale bar = 1 μm. (TIF) [file pgen.1009407.s008.tif]

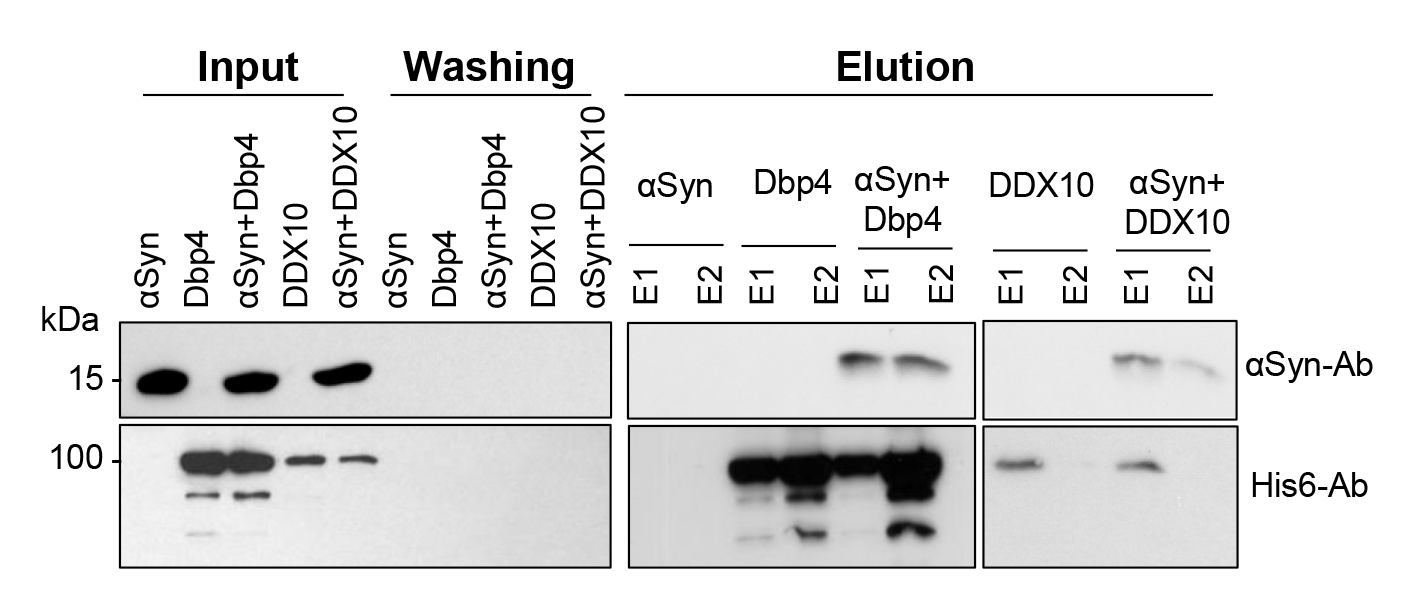

Supplement: S9 Fig — Dbp4 and DDX10 were expressed as 6xHis fusions in E. coli and purified. αSyn was purified without a tag. His6-tagged proteins were incubated alone or with αSyn for 30 min and subsequently bound to Ni-NTA beads. αSyn alone served as a control. After washing the samples were eluted. Western blot analysis was performed using αSyn and His6 antibodies with 10% of the input, 10% of the last washing fraction and 10% of the elution fraction 1 (E1) and elution fraction 2 (E2). (TIF) [file pgen.1009407.s009.tif]
