# Supplementary material for: DEAD-box RNA helicase Dbp4/DDX10 is an enhancer of α-synuclein toxicity and oligomerization
Source: PLoS Genet. 2021 Mar 3;17(3):e1009407. doi: 10.1371/journal.pgen.1009407 (PMC7928443; doi:10.1371/journal.pgen.1009407)
Supplement: S1 Table — The first column indicates the standard gene name. The second column indicates the human orthologs. Type and strength correspond to observed genetic interactions in spotting assays after downregulation of the essential genes resulting in synthetic-sick (-) phenotype (protective gene) or growth enhancement (+) phenotype (drug target). Brief description of the protein function is deduced from the Saccharomyces genome database (SGD). Genes were classified in one functional category according to their function or biological process using Gene Ontology (GO) annotations, generated by Saccharomyces Genome Database (SGD) Gene Ontology Slim Mapper, FunSpec webserver and manual curation. (DOCX) [file pgen.1009407.s010.docx]

**S1 Table. Identified genetic interactions between αSyn and *Tet*-alleles of essential genes.** The first column indicates the standard gene name. The second column indicates the human orthologs. Type and strength correspond to observed genetic interactions in spotting assays after downregulation of the essential genes resulting in synthetic-sick (**-**) phenotype (protective gene) or growth enhancement (**+**) phenotype (drug target). Brief description of the protein function is deduced from the *Saccharomyces* genome database (SGD). Genes were classified in one functional category according to their function or biological process using Gene Ontology (GO) annotations, generated by Saccharomyces Genome Database (SGD) Gene Ontology Slim Mapper, FunSpec webserver and manual curation.

| **GENE** | **HUMAN ORTHOLOG** | **TYPE / STRENGTH** | **FUNCTION** |
| --- | --- | --- | --- |
| **Transcription and RNA metabolism** | | | |
| *HSF1* | *HSF1* | **-** | Heat shock transcription factor |
| *CFT1* | *CPSF1* | **- -** | Pre-mRNA 3´-end processing factor |
| *RPB11* | *POLR2J* | **-** | DNA-directed RNA polymerase II subunit, 13.6 kD |
| *RPO26* | *POLR2F* | **- -** | DNA-directed RNA polymerase I, II, III, 18 kD subunit |
| *RPC40* | *POLR1C* | **- -** | DNA-directed RNA polymerase I, III, 40 kD subunit |
| *RPA43* | *-* | **+** | DNA-directed RNA polymerase I, 36 kD subunit |
| *RNA15* | *-* | **-** | Component of pre-mRNA 3´-end processing factor |
| **rRNA processing and ribosome biogenesis** | | | |
| *DBP4* | *DDX10* | ***+ +*** | Component of small subunit processome, required for small subunit biogenesis |
| *NOP4* | *RBM28* | **- -** | Nucleolar protein, essential for ribosomal subunit biogenesis |
| *RCL1* | *RCL1* | **-** | Component of small subunit processome, required for small subunit biogenesis |
| *RNT1* | *-* | **- -** | Double stranded ribonuclease (RNase III) required for pre-rRNA processing |
| **Cytoskeleton and Protein degradation** | | | |
| *RPN11* | *PSMD14* | **- -** | 26S proteasome regulatory subunit |
| *PRE5* | *PSMA1* | **- -** | 20S proteasome subunit (alpha6) |
| *CDC31* | *CETN3* | **-** | Involved in proteasomal protein degradation |
| *SPC110* | *PCNT* | ***+*** | Structural constituent of cytoskeleton |
| *TUB1* | *TUBA1C* | **- -** | Alpha-tubulin, structural constituent of cytoskeleton |
| **GTPase activator activity** | | | |
| *RNA1* | *CHADL* | ***+*** | Ran GTPase activator activity; involved in ribosomal subunit export from nucleus |
| *IQG1* | *IQGAP* | **- -** | Actin filament binding protein |
| **Others** | | | |
| *ERG29* | *-* | **-** | Possibly involved ergosterol biosynthesis |
| *HSP60* | *HSPD1* | **- -** | Heat shock protein - mitochondrial chaperone |
| *RET3* | *COPZ1* | **-** | Involved in retrograde transport between Golgi and ER |
