# Supplementary material for: DEAD-box RNA helicase Dbp4/DDX10 is an enhancer of α-synuclein toxicity and oligomerization
Source: PLoS Genet. 2021 Mar 3;17(3):e1009407. doi: 10.1371/journal.pgen.1009407 (PMC7928443; doi:10.1371/journal.pgen.1009407)
Supplement: S2 Table — The first column indicates the standard gene name; the second column indicates the systematic gene name. The third column gives a brief description of the protein’s function according to Saccharomyces genome database (SGD). “Score” represents the fitness of growth, calculated as ratio of the colony area of αSyn expressing strains to corresponding vector control on plates with galactose-inducing medium. The value is mean of four independent screens. The table lists hits with score < 0.85 and standard deviation < 0.05. Genes identified in both yTHC and DAmP screen are indicated by *. 76 out of 84 genes have human orthologs, indicated in the last column. (DOCX) [file pgen.1009407.s011.docx]

**S2 Table. Observed genetic interactions between αSyn and the hypomorphic** **alleles of essential genes from the DAmP collection.** The first column indicates the standard gene name; the second column indicates the systematic gene name. The third column gives a brief description of the protein's function according to *Saccharomyces* genome database (SGD). “Score” represents the fitness of growth, calculated as ratio of the colony area of αSyn expressing strains to corresponding vector control on plates with galactose-inducing medium. The value is mean of four independent screens. The table lists hits with score < 0.85 and standard deviation < 0.05. Genes identified in both yTHC and DAmP screen are indicated by *. 76 out of 84 genes have human orthologs, indicated in the last column.

| **GENE** | **SYSTEMATIC NAME** | **DESCRIPTION** | **SCORE** | **STDEV** | **HUMAN ORTHOLOG** |
| --- | --- | --- | --- | --- | --- |
| ***ABD1*** | YBR236C | mRNA cap methyltransferase | 0.80 | 0.04 | yes |
| ***APC4*** | YDR118W | Anaphase Promoting Complex | 0.79 | 0.05 | yes |
| ***ARH1*** | YDR376W | Mitochondrial protein putative ferredoxin-NADP+ reductase | 0.78 | 0.05 | yes |
| ***BDP1*** | YNL039W | RNA polymerase III recruiting transcription factor | 0.79 | 0.05 | yes |
| ***BET2*** | YPR176C | Required for ER to Golgi transport | 0.59 | 0.04 | yes |
| ***CDC19*** | YAL038W | Pyruvate kinase | 0.83 | 0.02 | yes |
| ***CDC3*** | YLR314C | Cell division control protein | 0.85 | 0.05 | yes |
| ***CDC33*** | YOL139C | Translation initiation factor eIF4E | 0.82 | 0.05 | yes |
| ***CDC37*** | YDR168W | Cell division control protein | 0.74 | 0.02 | yes |
| ***CDC39*** | YCR093W | Component of the CCR4-NOT1 core complex | 0.84 | 0.02 | yes |
| ***CFT1**** | YDR301W | Pre-mRNA 3´-end processing factor | 0.80 | 0.04 | yes |
| ***CWC2*** | YDL209C | Involved in mRNA splicing | 0.78 | 0.04 | no |
| ***DBF4*** | YDR052C | Regulatory subunit for Cdc7p protein kinase | 0.82 | 0.02 | yes |
| ***DCP1*** | YOL149W | mRNA DeCaPping | 0.76 | 0.04 | yes |
| ***DRS1*** | YLL008W | RNA-helicase of the DEAD box family involved in ribosome assembly | 0.81 | 0.02 | yes |
| ***DSN1*** | YIR010W | Important for chromosome segregation | 0.84 | 0.03 | yes |
| ***ERG10*** | YPL028W | Acetyl-CoA-acetyltransferase, cytosolic | 0.77 | 0.04 | yes |
| ***FCP1*** | YMR277W | tfiiF-associating component of Ctd Phosphatase | 0.82 | 0.02 | yes |
| ***GCD1*** | YOR260W | Gamma subunit of the translation initiation factor eIF2B | 0.81 | 0.01 | yes |
| ***GLE1*** | YDL207W | RNA export mediator | 0.84 | 0.04 | yes |
| ***GPI15*** | YNL038W | GlycosylPhosphatidylInositol anchor biosynthesis protein | 0.51 | 0.02 | yes |
| ***HEM1*** | YDR232W | 5-aminolevulinate synthase | 0.80 | 0.05 | yes |
| ***HEM12*** | YDR047W | Uroporphyrinogen decarboxylase | 0.82 | 0.03 | yes |
| ***HEM13*** | YDR044W | Coproporphyrinogen III oxidase | 0.84 | 0.04 | yes |
| ***HSF1**** | YGL073W | Heat Shock transcription Factor | 0.84 | 0.05 | yes |
| ***IQG1**** | YPL242C | Protein involved in cytokinesis | 0.77 | 0.02 | yes |
| ***IRA1*** | YBR140C | Inhibitory Regulator of the RAS-cAMP pathway | 0.84 | 0.03 | yes |
| ***KRS1*** | YDR037W | Lysyl (K) tRNA Synthetase | 0.79 | 0.04 | yes |
| ***LUC7*** | YDL087C | U1 snRNP protein with a role in 5´ splice site recognition | 0.82 | 0.02 | yes |
| ***MCM2*** | YBL023C | MiniChromosome Maintenance | 0.78 | 0.02 | yes |
| ***MPE1*** | YKL059C | 3' end formation of mRNA | 0.84 | 0.02 | yes |
| ***MTR3*** | YGR158C | Involved in mRNA decay | 0.85 | 0.04 | yes |
| ***NAT2*** | YGR147C | N-acetyltransferase for N-terminal methionine | 0.79 | 0.03 | yes |
| ***NHP2*** | YDL208W | Small nucleolar RNP-associated protein | 0.81 | 0.04 | yes |
| ***NMD3*** | YHR170W | Involved in nuclear export of the large ribosomal subunit | 0.83 | 0.03 | yes |
| ***NMT1*** | YLR195C | N-Myristoyl Transferase | 0.81 | 0.04 | yes |
| ***NOP14*** | YDL148C | Involved in maturation of small ribosomal subunits | 0.83 | 0.04 | yes |
| ***ORC2*** | YBR060C | Origin Recognition Complex | 0.80 | 0.04 | yes |
| ***PBN1*** | YCL052C | Protease B Non-derepressible | 0.83 | 0.04 | no |
| ***PCF11*** | YDR228C | Component of pre-mRNA 3'-end processing factor CF I | 0.84 | 0.02 | yes |
| ***PDI1*** | YCL043C | Protein Disulfide Isomerase | 0.83 | 0.05 | yes |
| ***POL12*** | YBL035C | DNA-directed DNA polymerase alpha, 70KD subunit | 0.79 | 0.03 | yes |
| ***PRE5**** | YMR314W | 20S proteasome subunit (alpha6) | 0.14 | 0.00 | yes |
| ***PTA1*** | YAL043C | Pre-tRNA processing protein / PF I subunit | 0.82 | 0.02 | yes |
| ***QRI1*** | YDL103C | UDP-N-acetylglucosamine pyrophosphorylase | 0.02 | 0.03 | yes |
| ***RFC5*** | YBR087W | DNA replication factor C, 40KD subunit | 0.84 | 0.02 | yes |
| ***RIO1*** | YOR119C | Kinase required for small ribosomal subunit assembly | 0.82 | 0.03 | yes |
| ***RIX7*** | YLL034C | Biogenesis of large ribosomal subunits | 0.76 | 0.04 | yes |
| ***RLI1*** | YDR091C | Protein promoting preinitiation complex assembly, required for large ribosomal subunit biogenesis | 0.81 | 0.03 | yes |
| ***RLP7*** | YNL002C | Nucleolar protein related to ribosomal protein L7 | 0.80 | 0.04 | yes |
| ***RNA14*** | YMR061W | Component of pre-mRNA 3'-end processing factor CF I | 0.81 | 0.03 | yes |
| ***RNA15**** | YGL044C | Component of pre-mRNA 3´-end processing factor | 0.81 | 0.04 | yes |
| ***RPB7*** | YDR404C | RNA polymerase II subunit B16 | 0.66 | 0.05 | yes |
| ***RPC11*** | YDR045C | RNA polymerase III subunit C11 | 0.77 | 0.05 | yes |
| ***RPN11**** | YFR004W | 26S proteasome regulatory subunit | 0.84 | 0.05 | yes |
| ***RPN5*** | YDL147W | 26S proteasome regulatory subunit | 0.79 | 0.04 | yes |
| ***RRN3*** | YKL125W | RNA polymerase I specific transcription factor | 0.82 | 0.05 | yes |
| ***RRN9*** | YMR270C | RNA polymerase I specific transcription initiation factor | 0.85 | 0.02 | no |
| ***RRP1*** | YDR087C | Processing rRNA precursors species to mature rRNAs | 0.80 | 0.04 | yes |
| ***RRP17*** | YDR412W | Exonuclease involved in pre-rRNA processing | 0.79 | 0.02 | yes |
| ***RRP7*** | YCL031C | Involved in ribosome biogenesis | 0.84 | 0.01 | yes |
| ***RSA4*** | YCR072C | WD-repeat protein involved in ribosome biogenesis | 0.85 | 0.03 | yes |
| ***SCC2*** | YDR180W | Sister Chromatid Cohesion | 0.82 | 0.01 | yes |
| ***SEC1*** | YDR164C | Protein transport protein | 0.84 | 0.03 | yes |
| ***SEC26*** | YDR238C | Coatomer complex beta chain of secretory pathway vesicles | 0.85 | 0.04 | yes |
| ***SEC5*** | YDR166C | Required for exocytosis | 0.85 | 0.05 | yes |
| ***SFI1*** | YLL003W | Function in budding yeast spindle pole body duplication | 0.79 | 0.01 | no |
| ***SNU56*** | YDR240C | U1 snRNP protein | 0.82 | 0.04 | no |
| ***SPC29*** | YPL124W | Spindle pole body component | 0.77 | 0.01 | no |
| ***SPC42*** | YKL042W | Spindle pole body component | 0.84 | 0.05 | no |
| ***SPP381*** | YBR152W | Essential protein present in native splicing complexes | 0.85 | 0.05 | no |
| ***SPT6*** | YGR116W | Transcription elongation protein | 0.83 | 0.04 | yes |
| ***SRP101*** | YDR292C | Signal recognition particle receptor, alpha chain | 0.83 | 0.01 | yes |
| ***SUA7*** | YPR086W | TFIIB subunit (transcription initiation factor), factor E | 0.85 | 0.04 | yes |
| ***TCP1*** | YDR212W | Mediates protein folding | 0.82 | 0.03 | yes |
| ***TFC6*** | YDR362C | Subunits of RNA polymerase III transcription initiation factor complex (TFIIIC) | 0.84 | 0.02 | yes |
| ***TIM22*** | YDL217C | TIM22 subunit of the TIM22 complex | 0.83 | 0.04 | yes |
| ***TSC13*** | YDL015C | ER protein involved in very long chain fatty acid synthesis | 0.81 | 0.03 | yes |
| ***TUB1**** | YML085C | Alpha-1-tubulin | 0.79 | 0.05 | yes |
| ***UTP5*** | YDR398W | U3 snoRNP-associated protein involved in ribosome biogenesis | 0.77 | 0.05 | yes |
| ***YAE1*** | YJR067C | Protection of ribosomal assembly | 0.84 | 0.05 | yes |
| ***YCG1*** | YDR325W | Yeast Condensin G | 0.78 | 0.04 | yes |
| ***YCS4*** | YLR272C | Yeast Condensin Subunit | 0.84 | 0.05 | yes |
| ***YHC1*** | YLR298C | Associated with the U1 snRNP complex | 0.79 | 0.03 | yes |
