# Supplementary material for: DEAD-box RNA helicase Dbp4/DDX10 is an enhancer of α-synuclein toxicity and oligomerization
Source: PLoS Genet. 2021 Mar 3;17(3):e1009407. doi: 10.1371/journal.pgen.1009407 (PMC7928443; doi:10.1371/journal.pgen.1009407)
Supplement: S3 Table — Genes were classified in one category according to their function or biological process using Gene Ontology (GO) annotations, generated by Saccharomyces Genome Database (SGD) Gene Ontology Slim Mapper, FunSpec webserver and manual curation. (DOCX) [file pgen.1009407.s012.docx]

**S3 Table. Distribution of the identified genes from DAmP screen into functional categories.** Genes were classified in one category according to their function or biological process using Gene Ontology (GO) annotations, generated by Saccharomyces Genome Database (SGD) Gene Ontology Slim Mapper, FunSpec webserver and manual curation.

| **BIOLOGICAL PROCESS** | **GENES** |
| --- | --- |
| Transcription and RNA metabolism | ABD1 BDP1 CDC39 CFT1 CWC2 DCP1 FCP1 HSF1 LUC7 MPE1 PCF11 PTA1 RNA14 RNA15 RPB7 RPC11 RRN3 RRN9 SNU56 SPP381 SPT6 SUA7 TFC6 YHC1 |
| rRNA processing and ribosome biogenesis | DRS1 MTR3 NHP2 NMD3 NOP14 RIO1 RLI1 RLP7 RRP1 RRP17 UTP5 RIX7 RSA4 RRP7 YAE1 |
| Cytoskeleton and protein degradation | CDC37 DSN1 IQG1 PBN1 PRE5 RPN11 RPN5 SFI1 SPC29 SPC42 TUB1 |
| Cell Cycle | APC4 CDC3 CDC33 DBF4 SCC2 YCS4 YCG1 |
| Golgi vesicle transport and secretion | BET2 SEC1 SEC5 SEC26 |
| Cofactor Metabolic Process | ARH1 HEM1 HEM12 HEM13 |
| Lipid Metabolic Process | ERG10 GPI15 TSC13 |
| Nucleobase-Containing Small Molecule Metabolic Process | IRA1 MCM2 QRI1 |
| DNA Replication | POL12 RFC5 ORC2 |
| Translational Initiation | GCD1 GLE1 |
| Protein Targeting | SRP101 TIM22 |
| Peptidyl-Amino Acid Modification | NAT2 NMT1 |
