# Supplementary material for: DEAD-box RNA helicase Dbp4/DDX10 is an enhancer of α-synuclein toxicity and oligomerization
Source: PLoS Genet. 2021 Mar 3;17(3):e1009407. doi: 10.1371/journal.pgen.1009407 (PMC7928443; doi:10.1371/journal.pgen.1009407)
Supplement: S4 Table — The subcellular localization and enrichment analysis were carried out using FunSpec webserver. (DOCX) [file pgen.1009407.s013.docx]

**S4 Table. Subcellular localization of the gene products of yTHC gene modifiers.** The subcellular localization and enrichment analysis were carried out using FunSpec webserver.

| **Category** | **In Category from Cluster** | **n** | **p-value** |
| --- | --- | --- | --- |
| nucleus | CFT1 RPN11 RNA15 HSF1 RNT1 PRE5 RPB11 RCL1 CDC31 RPA43 NOP4 RPC40 RPO26 | 13 | 0.002341 |
| nucleolus | DBP4 RNT1 RCL1 RPA43 NOP4 RPC40 RPO26 | 7 | 3.32e-05 |
| spindle pole body | SPC110 TUB1 CDC31 | 3 | 0.002089 |
| cytoskeleton | SPC110 TUB1 | 2 | 0.04472 |
