# Supplementary material for: DEAD-box RNA helicase Dbp4/DDX10 is an enhancer of α-synuclein toxicity and oligomerization
Source: PLoS Genet. 2021 Mar 3;17(3):e1009407. doi: 10.1371/journal.pgen.1009407 (PMC7928443; doi:10.1371/journal.pgen.1009407)
Supplement: S5 Table — The subcellular localization and enrichment analysis were carried out using FunSpec webserver. (DOCX) [file pgen.1009407.s014.docx]

**S5 Table. Subcellular localization of the gene products of DAmP screen modifiers.** The subcellular localization and enrichment analysis were carried out using FunSpec webserver.

| **Category** | **In Category from Cluster** | **n** | **p-value** |
| --- | --- | --- | --- |
| nucleus | PTA1 MCM2 POL12 ORC2 RFC5 SPP381 ABD1 CDC39 LUC7 QRI1 NOP14 GLE1 NHP2 CWC2 RPC11 HEM12 RRP1 RLI1 APC4 SCC2 PCF11 SNU56 CFT1 YCG1 TFC6 UTP5 RPB7 RRP17 RPN11 RNA15 HSF1 SPT6 MTR3 NMD3 DSN1 YAE1 SPC42 MPE1 RRN3 DRS1 RIX7 YCS4 YHC1 RNA14 RRN9 FCP1 PRE5 RLP7 BDP1 CDC33 RIO1 SUA7 | 52 | 8.39E-07 |
| nucleolus | RSA4 NOP14 NHP2 RRP1 UTP5 RRP17 MTR3 RRN3 DRS1 RIX7 YCS4 RRN9 RLP7 | 13 | 3.58E-05 |
| mRNA cleavage factor complex | PTA1 PCF11 CFT1 RNA15 RNA14 | 5 | 1.55E-02 |
| preribosome, large subunit precursor | RRP1 DRS1 RIX7 RLP7 | 4 | 0.0008093 |
| spliceosomal complex | SPP381 LUC7 CWC2 SNU56 | 4 | 0.001412 |
| mRNA cleavage and polyadenylation specificity factor complex | PTA1 CFT1 MPE1 | 3 | 0.00156 |
| U1 snRNP | LUC7 SNU56 YHC1 | 3 | 0.002247 |
| proteasome storage granule | RPN5 RPN11 PRE5 | 3 | 0.002321 |
| U2-type prespliceosome | LUC7 SNU56 YHC1 | 3 | 0.004181 |
| central plaque of spindle pole body | SPC42 SPC29 | 2 | 0.005173 |
| nuclear condensin complex | YCG1 YCS4 | 2 | 0.005769 |
| proteasome regulatory particle, lid subcomplex | RPN5 RPN11 | 2 | 0.006735 |
| commitment complex | SNU56 YHC1 | 2 | 0.008164 |
