# Supplementary material for: DEAD-box RNA helicase Dbp4/DDX10 is an enhancer of α-synuclein toxicity and oligomerization
Source: PLoS Genet. 2021 Mar 3;17(3):e1009407. doi: 10.1371/journal.pgen.1009407 (PMC7928443; doi:10.1371/journal.pgen.1009407)
Supplement: S6 Table — (DOCX) [file pgen.1009407.s015.docx]

**S6 Table. Reagents and Tools.**

| **Reagent/Resource** | **Reference or Source** | **Identifier or Catalog Number** |
| --- | --- | --- |

| Antibodies | | |
| --- | --- | --- |
| αSyn rabbit antibody | Santa Cruz Biotechnology | Cat#SC-10717 |
| αSyn rabbit antibody | AnaSpec | Cat#AS-53878 |
| GAPDH mouse antibody | ThermoFisher | Cat#MA5-15738 |
| Nop1 mouse antibody | Santa Cruz Biotechnology | Cat#SC-57940 |
| GFP rat antibody | Chromotek, Germany | Cat#3H9 |
| Syn1 mouse antibody | BD Transduction Laboratory, USA | Cat#610787 |
| Alexa Fluor 488 donkey anti-mouse | Life Technologies, USA | Cat# A-21202 |
| 6xHis mouse antibody | ThermoFisher | Cat# R930-25 |
| A11 rabbit antibody | Invitrogen | Cat# AHB0052 |
| Bacterial and Virus Strains | | |
| *Escherichia coli* DH5α | DSMZ, Germany | Cat#DSM 6897 |
| *Escherichia coli* BL21 (DE3) | DSMZ, Germany | Cat#DSM 25157 |
| Chemicals, Peptides, and Recombinant Proteins | | |
| Geneticin (G418) | Carl Roth, Germany | Cat#0239.4 |
| Canavanine | Enzo Life Sciences, USA | Cat# ALX-350-002-M100 |
| Thialysine | Sigma-Aldrich, Germany | Cat#A2636 |
| Doxycycline | Sigma-Aldrich, Germany | Cat#D9891 |
| Thioflavin T | Sigma-Aldrich, Germany | Cat#T3516 |
| Critical Commercial Assays | | |
| Yeast Nuclei Isolation Kit | BioVision, USA | Cat#K289 |
| QuikChange II Site-Directed Mutagenesis Kit | Agilent Technologies, USA | Cat#200523 |
| GeneArt Seamless Cloning and Assembly Kit | ThermoFisher, USA | Cat#A13288 |
| High Pure RNA Isolation Kit | Roche Diagnostics, Germany | Cat#11828665001 |
| QuantiTect Reverse Transcription Kit | Qiagen, Germany | Cat#205311 |
| Experimental Models: Cell Lines | | |
| Human Embryonic Kidney 293 cells | Sigma-Aldrich, Germany | Cat# 85120602 |
|  |  |  |
| Experimental Models: Organisms/Strains | | |
| Yeast Tet-Promoters Hughes Collection (yTHC) | Horizon Discovery, UK | Cat#YSC1182 |
| Yeast DAmP Library haploid | Horizon Discovery, UK | Cat#YSC5090 |
| R1158: *MATa his3-1 leu2-0 met15-0 URA3::CMV-tTA* | Horizon Discovery, UK | Cat#YSC1210 |
| Y7092: MAT*α* can1*Δ*::*STE2pr-Sp_his5 lyp1Δ* his3*Δ*1 ura3*Δ0* leu2*Δ*0 lys2+ met15*Δ0* | [1] | N/A |
| W303: *MATa; ura3-52; trp1D2; leu2-3_112; his3-11; ade2-1; can1-100* | ATCC | ATTC: 208353 |
| BY4741: *MATa; his3Δ 1; leu2Δ0; met15Δ0; ura3Δ0* | EUROSCARF | Y00000 |
| *MATa; ura3-52; trp1D2; leu2-3_112; his3-11; ade2-1; can1-100 GAL1::SNCA::GFP::URA3 (2 copies)* | [2] | RH3467 |
| *MATa; ura3-52; trp1D2; leu2-3_112; his3-11; ade2-1; can1-100 GAL1::SNCA::GFP::URA3 (3 copies)* | [2] | RH3468 |
| *MATa; ura3-52; trp1D2; leu2-3_112; his3-11; ade2-1; can1-100 GAL1::SNCA^A30P^::GFP::URA3 (3 copies)* | [2] | RH3471 |
| *MATα can1Δ::STE2pr-Sp_his5 lyp1Δ his3Δ1 ura3Δ0 lys2+ met15Δ0 GAL1::SNCA::GFP::LEU2 (2 copies)* | this work | RH3795 |
| *MATα can1Δ::STE2pr-Sp_his5 lyp1Δ his3Δ1 ura3Δ0 lys2+ met15Δ0 GAL1-empty::LEU2* | this work | RH3796 |
| *MATa his3-1 leu2-0 met15-0 DBP4::kanR-tet07-TATA URA3::CMV-tTA GAL1::SNCA::GFP::LEU2 (2 copies)* | this work | RH3798 |
| *MATa his3-1 leu2-0 met15-0 DBP4::kanR-tet07-TATA URA3::CMV-tTA GAL1::SNCA::GFP::LEU2 (3 copies)* | this work | RH3799 |
| YJL033W: *DBP4-GFP* | Invitrogen Yeast GFP collection | Cat#95702 |
| YPL043W: *NOP4-GFP* | Invitrogen Yeast GFP collection | Cat#95702 |
| YOL010W: *RCL1-GFP* | Invitrogen Yeast GFP collection | Cat#95702 |
| Recombinant DNA | | |
| cDNA *DDX10* | BioCat | Cat#BC091521-TCH1003-GVO-TRI |
| *2µm; HIS3; GAL1pr; CYC1term; AmpR* | [3] | p423-GAL1 |
| *2µm; LEU2; GAL1pr; CYC1term; AmpR* | [3] | p425-GAL1 |
| *2µm; URA3, GAL1pr; CYC1term; AmpR* | [3] | p426-GAL1 |
| *LEU2; GAL1pr; CYC1term; AmpR* | [4] | pRS305 |
| *p426-GAL1::GFP* | [2] | pME3759 |
| *p426-GAL1::SNCA* | [2] | pME3760 |
| *p426-GAL1::SNCA::GFP* | [2] | pME3763 |
| *p426-GAL1::SNCA^A30P^::GFP* | [2] | pME3764 |
| *p426-GAL1::SNCA::mCherry* | [2] | pME3772 |
| *p423-GAL1::SNCA::VenusC* | [5] | N/A |
| *p426-GAL1::VenusN::SNCA* | [5] | N/A |
| *p423-GAL1::VenusC* | this study | pME5035 |
| *p426-GAL1::VenusN::DBP4* | this study | pME5036 |
| *p426-GAL1::VenusN* | this study | pME5090 |
| *p423-GAL1::DBP4::VenusC* | this study | pME5091 |
| *pUN100-Nop1-mRFP* | [6] | N/A |
| *pRS305 with TRP1* | this study | pME5037 |
| *pBP10-GAL1::SNCA::GFP* | this study | pME5038 |
| *p425-GAL1::SNCA::GFP* | this study | pME5039 |
| *p423-GAL1::DBP4* | this study | pME5041 |
| *p423-GAL1::Nop4* | this study | pME5042 |
| *p423-GAL1::Rcl1* | this study | pME5043 |
| *p423-GAL1::DBP4^K91R^* | this study | pME5044 |
| *p423-GAL1::DBP4^S225A/T227A^* | this study | pME5045 |
| *p423-GAL1::DBP4::GFP* | this study | pME5046 |
| *pET22b-DBP4::His6* | this study | pME5047 |
| *p423-GAL1::DDX10* | this study | pME5048 |
| *pET22b-DDX10::His6* | this study | pME5049 |
| *pET22b-SNCA* | this study | pME4913 |
| *pcDNA3.1-VenusN::DDX10* | this study | pME5050 |
| *pcDNA3.1-DDX10::mCherry* | this study | pME5051 |
| *pcDNA3.1-SNCA::VenusC* | [7] | N/A |
| *pcDNA3.1-VenusN::SNCA* | [7] | N/A |
| Software and Algorithms | | |
| ImageJ | [8] | https://imagej.nih.gov/ij/ |
| GraphPad Prism | GraphPad Software | N/A |
| Balony | [9] | http://barrypyoung.github.io/balony/ |
| Saccharomyces Genome Database (SGD) | [10] | www.yeastgenome.org |
| FunSpec | [11] | http://funspec.med.utoronto.ca/ |
| Other |  |  |
| ROTOR HDA bench robot | Singer Instruments, UK | N/A |
| PlusPlates | Singer Instruments, UK | N/A |
| Replicator pads | Singer Instruments, UK | N/A |
| CFX Connect Real-Time System | BioRad Laboratories, Germany | N/A |
| Tecan Infinite M200 | Tecan, Switzerland | N/A |

**References**

1. Tong AHY, Boone C. 16 High-Throughput Strain Construction and Systematic Synthetic Lethal Screening in Saccharomyces cerevisiae. Methods in Microbiology. Academic Press Inc.; 2007. pp. 369–707. doi:10.1016/S0580-9517(06)36016-3

2. Petroi D, Popova B, Taheri-Talesh N, Irniger S, Shahpasandzadeh H, Zweckstetter M, et al. Aggregate clearance of alpha-synuclein in Saccharomyces cerevisiae depends more on autophagosome and vacuole function than on the proteasome. J Biol Chem. 2012/06/23. 2012;287: 27567–27579. doi:M112.361865 [pii]10.1074/jbc.M112.361865

3. Mumberg D, Muller R, Funk M, Müller R, Funk M. Regulatable promoters of Saccharomyces cerevisiae: comparison of transcriptional activity and their use for heterologous expression. Nucleic Acids Res. 1994/12/25. 1994;22: 5767–8.

4. Sikorski RS, Hieter P. A system of shuttle vectors and yeast host strains designed for efficient manipulation of DNA in Saccharomyces cerevisiae. Genetics. 1989;122: 19–27.

5. Tenreiro S, Rosado-Ramos R, Gerhardt E, Favretto F, Magalhães F, Popova B, et al. Yeast reveals similar molecular mechanisms underlying alpha- and beta-synuclein toxicity. Hum Mol Genet. 2016;25: 275–290. doi:10.1093/hmg/ddv470

6. Heininger AU, Hackert P, Andreou AZ, Boon KL, Memet I, Prior M, et al. Protein cofactor competition regulates the action of a multifunctional RNA helicase in different pathways. RNA Biol. 2016;13: 320–330. doi:10.1080/15476286.2016.1142038

7. Lázaro DF, Rodrigues EF, Langohr R, Shahpasandzadeh H, Ribeiro T, Guerreiro P, et al. Systematic Comparison of the Effects of Alpha-synuclein Mutations on Its Oligomerization and Aggregation. PLoS Genet. 2014;10: e1004741. doi:10.1371/journal.pgen.1004741

8. Schneider CA, Rasband WS, Eliceiri KW. NIH Image to ImageJ: 25 years of image analysis. Nature Methods. 2012. pp. 671–675. doi:10.1038/nmeth.2089

9. Young BP, Loewen CJ. Balony: a software package for analysis of data generated by synthetic genetic array experiments. BMC Bioinformatics. 2013/12/07. 2013;14: 354. doi:10.1186/1471-2105-14-354

10. Cherry JM, Hong EL, Amundsen C, Balakrishnan R, Binkley G, Chan ET, et al. Saccharomyces Genome Database: The genomics resource of budding yeast. Nucleic Acids Res. 2012;40. doi:10.1093/nar/gkr1029

11. Robinson MD, Grigull J, Mohammad N, Hughes TR. FunSpec: A web-based cluster interpreter for yeast. BMC Bioinformatics. 2002;3. doi:10.1186/1471-2105-3-35
